# Multi-omics analysis and functional validation reveal the oncogenic role of TRIP13

**DOI:** 10.3389/fimmu.2026.1691436

**Published:** 2026-04-22

**Authors:** Yuanqiao Zhao, Yongqi Zhao, Ruilin Liu, Jing Li, Yinhuai Wang

**Affiliations:** 1 Department of Urology, The Second Xiangya Hospital, Central South University, Changsha, Hunan, China; 2 Department of Rehabilitation, The Second Xiangya Hospital, Central South University, Changsha, Hunan, China

**Keywords:** immune infiltration, palbociclib resistance, pan-cancer, prognosis, prostate cancer, TRIP13

## Abstract

**Background:**

Thyroid hormone receptor-interacting protein 13 (TRIP13), an enzyme from the AAA-ATPase family, facilitates the assembly or disassembly of protein complexes and participates in various biological processes. However, its impact on cancer immune infiltration and pan-cancer prognosis remains largely unexplored.

**Methods:**

Pan-cancer multi-omics data from publicly available resources were systematically analyzed to evaluate TRIP13 expression across various cancer types and its association with patient prognosis. In addition, functional enrichment analyses were conducted to investigate TRIP13-related biological processes and pathways. The analysis included GSEA enrichment, correlation with immune regulator expression, tumor immune cell infiltration, association with tumor mutational burden (TMB), and correlation with microsatellite instability (MSI). Additionally, single-cell data were used to explore the expression and potential role of TRIP13 at the single-cell level. We subsequently conducted a series of *in vitro* experiments.

**Results:**

Our comprehensive pan-cancer analysis reveals significantly elevated TRIP13 expression across multiple cancer types and links it to poor prognostic outcomes. TRIP13 primarily activates pathways such as ubiquitination, cell cycle regulation, and DNA repair to drive tumor progression. Additionally, TRIP13 expression exhibits complex associations with various immune regulators and immune cells. In prostate cancer, TRIP13 shows marked overexpression and is associated with unfavorable prognosis. We identified a significant upregulation of TRIP13 in proliferative tumor stem-like populations in prostate cancer. Consistently, prostate cancer cells that acquired resistance to CDK4/6 inhibitors displayed marked TRIP13 overexpression, and functional assays revealed that TRIP13 modulates cellular sensitivity to these agents. Mechanistically, we demonstrated that E2F1 transcriptionally activates TRIP13, which in turn drives the upregulation of the downstream ubiquitin ligase HECTD3.

**Conclusion:**

This study reveals aberrant TRIP13 expression across multiple cancers and its association with immune modulation and tumor aggressiveness. The elevation of TRIP13 in palbociclib resistant prostate cancer, together with the regulatory E2F1–TRIP13–HECTD3 axis, highlights its potential as a prognostic biomarker and therapeutic target.

## Introduction

Cancer represents a major social, public health, and economic challenge in the 21st century, responsible for one in six (16.8%) global deaths and one in four (22.8%) deaths from non-communicable diseases (NCDs) (1). Cancer treatment primarily relies on conventional methods such as surgery, chemotherapy, radiotherapy, and targeted therapy. Despite significant progress in recent years, many tumors, including prostate cancer, remain incurable due to the absence of highly effective therapeutic targets and the challenges posed by drug resistance. Prostate cancer exhibits both high incidence and mortality rates (1–3). Early-stage prostate cancer typically responds well to active treatment and has a relatively favorable prognosis (4). However, advanced prostate cancer often progresses rapidly to the castration-resistant prostate cancer (CRPC) stage following endocrine therapy (5–8), which signifies a poorer prognosis. This underscores the urgent need for new and reliable therapeutic targets to improve early detection, diagnosis, and treatment.

TRIP13, a member of the AAA (ATPase associated with various cellular activities) ATPase family, influences cell signaling by modifying substrate macromolecule conformations and participates in numerous cellular biological processes (9). TRIP13 is essential for meiotic recombination, chromosome synapsis, DNA repair, cell cycle progression, and the spindle assembly checkpoint (10, 11). Recent studies increasingly suggest that TRIP13 functions as an oncogene in tumors. A study indicates that TRIP13 contributes to radiation therapy resistance in head and neck cancer (11). Other studies have reported that high TRIP13 expression contributes to disease progression in multiple myeloma (9), colorectal cancer (12), and hepatocellular carcinoma (13). Previous studies identified TRIP13 as a pro-oncogenic factor in prostate cancer, primarily in early-stage disease (14). In contrast, major clinical challenges revolve around CRPC, and effective interventions for end-stage prostate cancer remain unavailable. CDK4/6 inhibitors offer a promising therapeutic avenue, yet their clinical impact remains modest. Moreover, the field lacks a systematic pan-cancer evaluation of TRIP13. To close this knowledge gap, we conducted a comprehensive pan-cancer investigation and delineated the contribution of TRIP13 to CDK4/6 inhibitor resistance in prostate cancer.

We found that TRIP13 displays broad overexpression across multiple malignancies and correlates with an unfavorable prognosis. TRIP13 shows strong associations with various immune regulatory features, suggesting a potential role in shaping tumor immune interactions. In prostate cancer, TRIP13 exhibits marked upregulation and is linked to poorer prognosis as well as several adverse pathological characteristics. TRIP13 reduces the sensitivity of prostate cancer cells to palbociclib. Mechanistically, E2F1 transcriptionally activates TRIP13, and TRIP13 enhances the expression of the downstream ubiquitin ligase HECTD3, thereby diminishing cellular responsiveness to palbociclib.

## Materials and methods

### Data collection and processing

Transcriptomic data and 33 types of cancer clinical profiles were retrieved from the UCSC Xena database (https://xena.ucsc.edu/). Data from the Cancer Cell Line Encyclopedia (CCLE; 23Q2 release) were obtained through the DepMap database (https://depmap.org/portal/). The mRNA expression data of TRIP13 in human normal tissues were obtained from the Genotype Tissue Expression (GTEx) database (https://gtexportal.org/home/). Protein levels of TRIP13 across various cancer types were analyzed using data from the Clinical Proteomic Tumor Analysis Consortium (CPTAC) (https://ualcan.path.uab.edu/analysis-prot.html). Additionally, RNA-seq and microarray expression profiles together with clinical annotations from 18 independent prostate cancer cohorts were retrieved from the Gene Expression Omnibus (GEO) and related public repositories, including GSE53922 (*n* = 112), GSE70770 (*n* = 293), GSE46602 (*n* = 50), PRAD_SU2C_2019 (*n* = 266), DKFZ2018 (*n* = 118), MSKCC2010 (*n* = 156), GSE70769 (*n* = 94), CHPCMA (*n* = 247), GSE32269 (*n* = 51), GSE74367 (*n* = 56), GSE66187 (*n* = 95), GSE80609 (*n* = 45), GSE101607 (*n* = 48), GSE21034 (*n* = 185), GSE32571 (*n* = 98), GSE71016 (*n* = 95), GSE69223 (*n* = 30), and GSE16560 (*n* = 281), along with shTRIP13 RNA-seq data from GSE109029. The datasets MSKCC2010, PRAD_SU2C_2019, and DKFZ2018 were retrieved from the cBioPortal for Cancer Genomics (https://www.cbioportal.org/). The CHPCMA data were referenced in this article (15).

### Spatial transcriptomic and single-cell RNA sequencing analysis

Single-cell transcriptome data from GSE206962 and GSE137829, comprising one castration-sensitive prostate cancer (CSPC) dataset and CRPC datasets, were retrieved from the GEO database and filtered using the following criteria: nFeature > 200, nFeature < 4000, and mitochondrial content < 10%. Data were analyzed using the R package Seurat (4.4.0) (16). To eliminate batch effects, data integration was performed using the Harmony R package (17). We analyzed publicly available spatial transcriptomic data, including human brain metastases from GSE179572, renal cancer from GSE175540, prostate cancer from GSE308486 and colorectal cancer with liver metastases from reference (18).

### TRIP13 activity score

The top 100 genes most correlated with TRIP13 in cancer were used as a gene set for enrichment analysis the GSVA R package, generating the TRIP13 activity score.

### Survival analysis

One-way Cox regression analysis were applied to the TCGA dataset, utilizing the “survival” and “forestplot” R packages, evaluated the correlation between TRIP13 expression and tumor prognosis, including overall survival (OS), disease-specific survival (DSS), disease-free survival (DFS), and progression-free survival (PFS). Kaplan–Meier survival analysis evaluated clinical outcomes in patients with low or high TRIP13 expression, conducted using the “survival” and “survminer” R packages.

### Correlation analysis of TRIP13 with the tumor immune microenvironment

The relative abundance of various cells in the tumor microenvironment was assessed using the CIBERSORT method, implemented via the CIBERSORT function. Additionally, the ESTIMATE tool evaluated tumor purity and stromal and immune scores (19).

### Gene set enrichment analysis

Patients in each TCGA cancer type were grouped into high and low TRIP13 expression levels, and differentially expressed genes underwent GSEA enrichment analysis. Cancer related hallmark gene set files were obtained from the msigdbr R package. The normalized enrichment score (NES) and *p*-values were calculated for differentially expressed genes (DEGs) using GSEA. Results were summarized and visualized as a bubble plot with the ggplot2 R package.

### Correlation analysis

TMB and MSI scores were retrieved from The Cancer Genome Atlas database (https://portal.gdc.cancer.gov/), and Spearman’s rank method was applied to assess the correlation between TRIP13 and TMB/MSI. The results were visualized using radar maps. Immune-related regulators were sourced from this article (20). Correlation analysis between TRIP13 expression and immune-related regulators across cancer types was performed using the cor.test function with TCGA data, and the results were visualized using ggplot2.

### Cell lines and cell culture

The prostate cancer cell lines PC-3 (SC0126) and C4-2 (SNL-160) were purchased from Yuchicell Biology Technology (Shanghai, China) and Wuhan Shang En Biotechnology Co., Ltd (Wuhan, China), respectively. Short tandem repeat (STR) profiling was performed to authenticate the cell lines, and mycoplasma contamination was regularly screened using the PlasmoTest Mycoplasma Detection Kit (Catalog #: rep-pt1, InvivoGen, China). The cells were cultured in RPMI-1640 media (Gibco, USA) supplemented with 10% fetal bovine serum (FBS) (Catalog #: AC03L055, Shanghai Life-iLab Biotech, China) and maintained at 37°C in a humidified atmosphere containing 5% CO_2_.

### Chemicals and reagents

The following primary antibodies were used for immunoblotting: Flag-Tag (#20543-1-AP, Proteintech, Wuhan, China; 1:20,000), GAPDH (#60004-1-Ig Proteintech, Wuhan, China; 1:50,000), TRIP13 (#19602-1-AP Proteintech, Wuhan, China; 1:5,000), E2F1 (#12171-1-AP Proteintech, Wuhan, China; 1:1,000), HECTD3 (#11487-1-AP Proteintech, Wuhan, China; 1:2,000), and cleaved caspase 3 (#25128-1-AP Proteintech, Wuhan, China; 1:1,000). Palbociclib HCl (#S1116) was purchased from Selleck (Shanghai, China). The sources of all the other reagents are explicitly indicated in their respective sections.

### Plasmid construction and RNAi reagents

OmicLink™ Expression Clone (CMV Promoter) plasmids (Catalog #: EX-V0006-M14, GeneCopoeia, USA) were utilized to clone the cDNA of TRIP13 and E2F1 for their overexpression. The sequences of the shRNA targeting TRIP13, E2F1, and HECTD3, obtained from RiboBio (Guangzhou, China), are listed in **Supplementary Table 1**.

### Quantitative real-time PCR analysis

Total RNA was extracted from cells using the TRIzol reagent (#AG21102, Accurate Biotechnology, Hunan, China). Reverse transcription kits (#AG11728, Accurate Biotechnology, Hunan, China) and PCR kits (#AG11701, Accurate Biotechnology, Hunan, China) were used for RT-qPCR according to the manufacturer’s instructions. Data were normalized to GAPDH values, and the fold change was quantified using the 2^−ΔΔCt^ method. The primer sequences are provided in **Supplementary Table 2**.

### Plasmid and shRNA transfection

After plating and serum starvation for 12 h in serum-free Opti-MEM medium (Catalog #: 31985062, Thermo Fisher Scientific, Shanghai, China), the designated plasmids or shRNAs were mixed with Lipofectamine 2000 (#12566014, Thermo Fisher Scientific, Shanghai, China) and incubated for 20 min in 1 mL of serum-free Opti-MEM medium. The mixture was then added to the plates or dishes. After 6 h, the medium was replaced with complete RPMI-1640, and cells were incubated for 24 h (plasmids) or 72 h (shRNAs). Puromycin selection was applied to isolate positively transfected cells.

### Chromatin immunoprecipitation and ChIP-qPCR

Following the manufacturer’s instructions, the chromatin immunoprecipitation (ChIP) Kit Magnetic - One Step (Abcam, ab156907, USA) and the chromatin extraction kit (Abcam, ab117152, USA) were used for ChIP-qPCR. Immunoprecipitation was performed using the appropriate antibodies as follows: E2F1 antibody (#66515-1-Ig, Proteintech, Wuhan, China, 1:50 dilution) for the ChIP experiment. Rabbit IgG antibody (#3900S, Cell Signaling Technology, Boston, America, 1:1,000 dilution) and mouse IgG antibody (#61656S, Cell Signaling Technology, Boston, America, 1:1,000 dilution) were used as negative controls. Primers were designed based on the promoter sequences of the target genes. The ChIP-qPCR primer sequences are provided in **Supplementary Table 3**.

### Dual-luciferase reporter assay

Cells were seeded in 24-well plates at a density of 50,000 cells per well and cultured for 24 h. The cells were then co-transfected with a firefly luciferase reporter plasmid (pGL3-Basic, Promega, Madison, America) containing the target gene promoter and a Renilla luciferase control plasmid (pRL-SV40, Promega, Madison, America) using liposome-based transfection. After 48 h, firefly and Renilla luciferase activities were measured sequentially with a multifunctional microplate reader. Relative luciferase activity was calculated by normalizing firefly luminescence to Renilla luminescence.

### *In vitro* cell proliferation assay

Cell proliferation was assessed using the Cell Counting Kit-8 (CCK-8, #C0037, Beyotime, Shanghai, China) according to the manufacturer’s instructions. Briefly, PC-3 and C4–2 cells were seeded in 96-well plates at a density of 1,000 cells per well in 100 µL of complete medium (with 10% FBS) for 24 h. After treatment under different conditions, 10 µL of CCK-8 reagent was added to each well and incubated for 1 h. The absorbance at 450 nm was then measured using a microplate reader.

### Colony formation assays

In the colony formation assay, cells from each experimental group were harvested, counted, and adjusted to a uniform density. Equal numbers of cells were then seeded into 6-well plates and cultured for 12 days. Subsequently, the cells were fixed with 4% paraformaldehyde and stained with crystal violet. Colonies, defined as clusters containing at least 50 cells, were counted manually, and the results were quantified for statistical analysis.

### Western blot analysis

Proteins were separated by sodium dodecyl sulfate-polyacrylamide gel electrophoresis (SDS-PAGE) in preparation for Western blot analysis. The separated proteins were transferred to 0.45-µm polyvinylidene fluoride (PVDF) membranes (Millipore, USA) and incubated sequentially with primary antibodies and corresponding secondary antibodies. Protein signals were detected using the ChemiDoc XRS system (Bio-Rad Laboratories, USA) after incubation with ECL detection reagent (Thermo Fisher Scientific, USA).

### Apoptosis assay

The Annexin V-FITC/PI assay and the caspase-3 activity assay were used to evaluate cell apoptosis. The caspase-3 activity assay was performed using the Caspase-3 Assay Kit (ab39401, Abcam) according to the manufacturer’s instructions. The Annexin V-FITC/PI Apoptosis Detection Kit (Solarbio Life Science, Beijing, China) was employed for the Annexin V-FITC/PI assay. Following the kit’s instructions, cells were stained with Annexin V-FITC and PI, incubated at room temperature for 15 min, and then analyzed using a flow cytometer (FACSCalibur, Becton, Dickinson and Company, USA). The FlowJo software was used for data analysis and interpretation.

### Statistical analysis

Statistical differences between the two groups were evaluated using the Student’s *t*-test or the Wilcoxon test, depending on data distribution. Differences among multiple groups were analyzed using one-way ANOVA or the Kruskal–Wallis test with appropriate *post-hoc* comparisons. The chi-square test assessed categorical variables. Univariate Cox regression and Kaplan–Meier methods evaluated the prognostic value of TRIP13 levels. Pearson or Spearman correlation tests measured relationships between variables based on data conditions. Receiver-operating characteristic (ROC) analysis determined the specificity and sensitivity of candidate indicators, with the area under the curve (AUC) calculated for diagnostic biomarkers. All statistical analyses were conducted using RStudio and GraphPad software. *p*-value <0.05 was considered statistically significant.

## Results

### Expression patterns of TRIP13 in normal and cancer tissues

**Figure 1:**
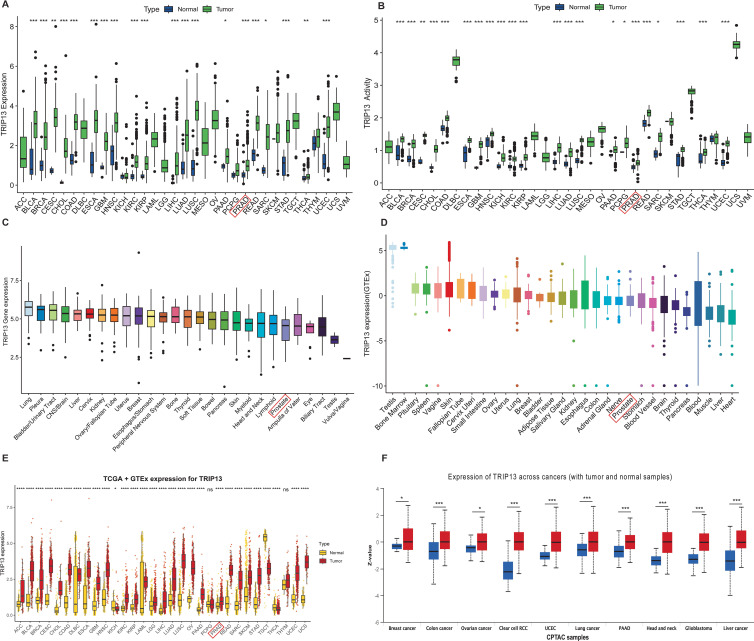
mRNA/protein expression levels of TRIP13 in human normal and tumor tissues. **(A)** Expression levels of TRIP13 in tumor (green) and normal (blue) tissues in the TCGA database. **(B)** TRIP13 activity scores in tumor (green) and normal (blue) tissues. **(C, D)** TRIP13 expression across various cell lines in DepMap datasets **(C)** and different tissues in GTEx datasets **(D)**. **(E)** TRIP13 expression in the integrated TCGA and GTEx datasets (normal: yellow; tumor: red). **(F)** TRIP13 protein expression in tumor (red) and normal (blue) tissues across cancers in CPTAC samples. * indicates *p* < 0.05; ** indicates *p* < 0.01; *** indicates *p* < 0.001.

In the TCGA database, TRIP13 exhibited high expression in most tumor tissues compared to normal tissues, including PRAD, BLCA, and COAD, while no significant differences were observed in KICH, PCPG, SKCM, and THYM (**Figure 1A**). TRIP13 activity scores were also found to be generally elevated in most tumors (**Figure 1B**). In the DepMap database, TRIP13 expression reached the highest levels in lung cancer and showed relatively low levels in prostate cancer (**Figure 1C**). In the GTEx database, TRIP13 expression appeared highest in the testis and bone marrow, while the lowest levels occurred in the heart and liver (**Figure 1D**). TRIP13 is predominantly overexpressed in tissues with elevated cell proliferation and differentiation. After integrating data from the TCGA and GTEx databases, TRIP13 displayed elevated expression in most tumors (**Figure 1E**), a trend further confirmed at the protein level (**Figure 1F**). Overall, TRIP13 expression displayed an increase in tumors but remained relatively low in normal tissues. TRIP13 expression was notably upregulated in tumor tissues, prompting further exploration of its variation across different clinicopathological subgroups. Significant differences in TRIP13 expression were observed across age groups in tumors such as BRCA, ESCA, HNSC, LUAD, LUSC, and PRAD (**Supplementary Figure 1A**). Gender-related differences in TRIP13 expression were identified in tumors such as HNSC and LAML (**Supplementary Figure 1B**). In tumors such as BLCA, CESC, HNSC, and KIRC, TRIP13 expression levels rose with advancing grades (**Supplementary Figure 1C**). Similarly, elevated TRIP13 expression correlated with advanced stages in tumors such as ACC, BLCA, LUAD, and KIRC (**Supplementary Figure 1D**). These findings suggest that TRIP13 may function as an oncogene in various tumors.

### Spatial localization and cluster-specific expression of TRIP13

**Figure 2:**
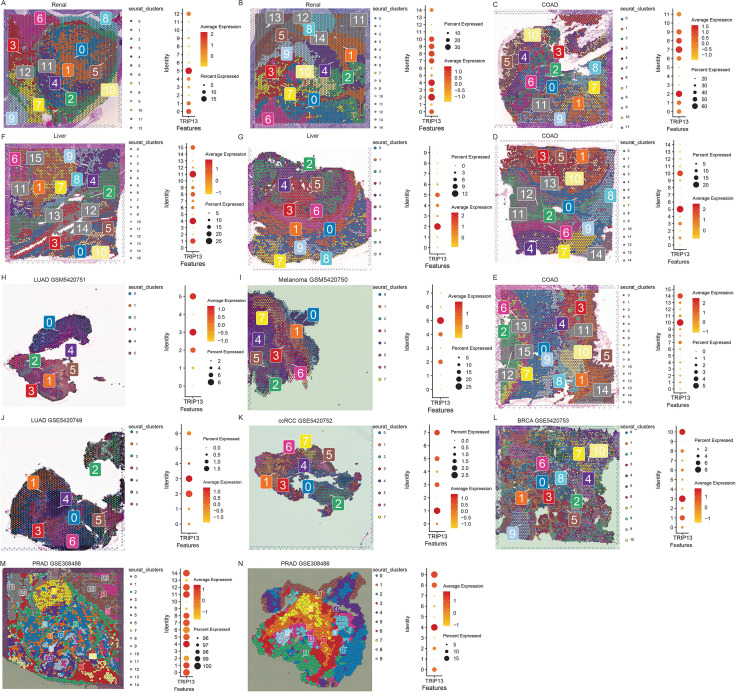
Spatial transcriptomic profiling of TRIP13 across multiple tumor types. **(A, B)** Renal cancer, **(C–E)** colorectal cancer, **(F, G)** colorectal cancer liver metastases, and **(H–L)** brain metastases derived from distinct primary tumors and **(M, N)** prostate cancer. Analyses performed using the Seurat-based spatial clustering. Left panels: Spatial maps highlighting cluster-specific segmentation of tissue sections. Right panels: Dot plots showing the average TRIP13 expression and the percentage of TRIP13-positive cells in each cluster.

Given the constraints in data availability and journal space, we performed spatial transcriptomic analysis in renal cancer (**Figures 2A, B**), colorectal cancer (**Figures 2C–E**), and colorectal cancer liver metastases (**Figures 2F, G**) and brain metastasis tissues originating from lung adenocarcinoma, melanoma, clear cell renal cell carcinoma, breast cancer (**Figures 2H–L**), and prostate cancer (**Figures 2M, N**). Across all tumor types, TRIP13 showed markedly elevated expression and preferential localization within tumor-associated clusters.

### Prognostic significance of TRIP13 in human cancers

**Figure 3:**
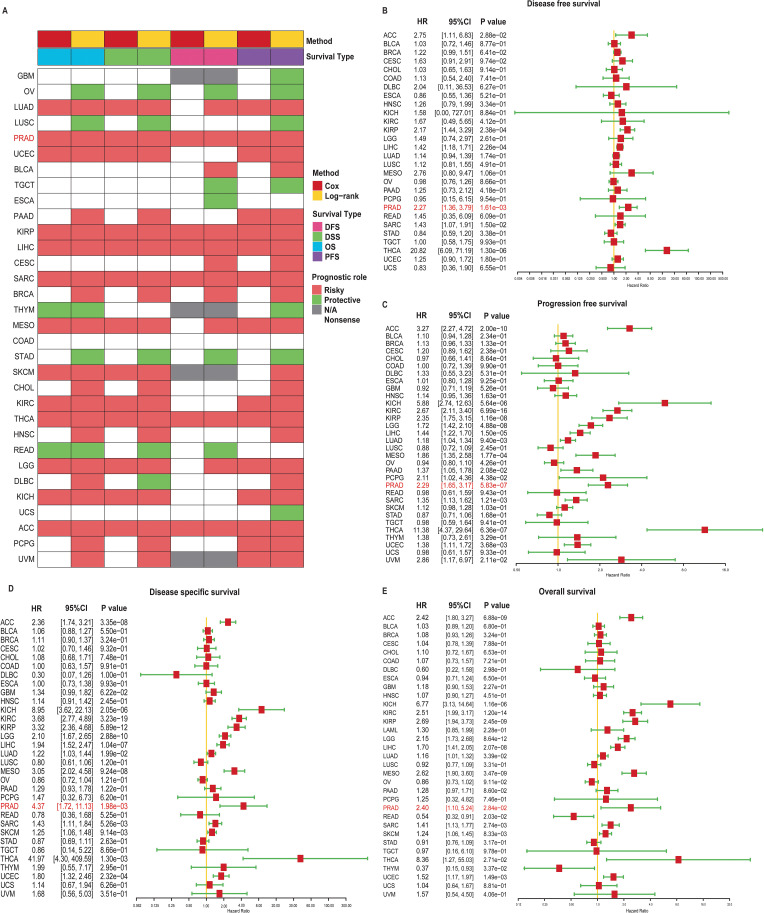
Comprehensive survival analysis of TRIP13 in cancer. **(A)** Summary of TRIP13’s prognostic roles in pan-cancer. Each row represents a specific cancer type, colored by the analysis method (Cox or log-rank), survival type (DFS, DSS, OS, or PFS), and prognostic role (risky or protective). Gray cells indicate that the data are not available. White boxes represent the analyses that are not significant. **(B)** Disease-free survival (DFS). **(C)** Progression-free survival (PFS). **(D)** Disease-specific survival (DSS). **(E)** Overall survival (OS). The forest plot shows hazard ratios (HRs), 95% confidence intervals (CIs), and *p*-values for TRIP13 expression in relation to DFS, PFS, DSS, and OS across multiple cancer types. Each red square represents the HR, and horizontal lines indicate the 95% CI. Yellow vertical lines mark HR = 1.

To evaluate the prognostic impact of TRIP13 mRNA levels in human cancers, curated survival data, including OS, DSS, DFS, and PFS for various cancer types, were downloaded and analyzed from the UCSC database. Heatmap revealed that patients with high TRIP13 expression generally exhibited worse prognoses across multiple tumor types, including PRAD, KIRP, LIHC, THCA, and ACC (**Figure 3A**). We then present forest plots illustrating the prognostic differences across various tumor types. In DFS, TRIP13 functioned as a risk factor for ACC, KIRP, LIHC, PRAD, SARC, and THCA, with no statistically significant differences observed in other tumors (**Figure 3B**). In PFS, TRIP13 acted as a risk factor for ACC, KICH, LIHC, LUAD, MESO, PAAD, PCPG, and PRAD (**Figure 3C**). In DSS, TRIP13 served as a risk factor for KICH, KIRC, KIRP, LGG, LIHC, and PRAD (**Figure 3D**). In OS, TRIP13 was identified as a risk factor for LGG, LUAD, and PRAD (**Figure 3E**). Notably, TRIP13 consistently appeared as a risk factor in ACC, PRAD, LIHC, and THCA across multiple survival analyses. However, its association lacked consistency in other tumor types. We generated Kaplan–Meier survival curves for DFS, PFS, DSS, and OS across various tumors. High TRIP13 expression correlated with poor prognosis in most malignancies, including PRAD and KIRC. Conversely, in tumors such as OV and READ, higher TRIP13 expression was associated with improved prognosis (**Supplementary Figures 2–5**). Taken together, these results demonstrate that TRIP13 expression serves as an effective prognostic factor across multiple cancers.

### GSEA analysis of TRIP13 functions in human cancers

**Figure 4:**
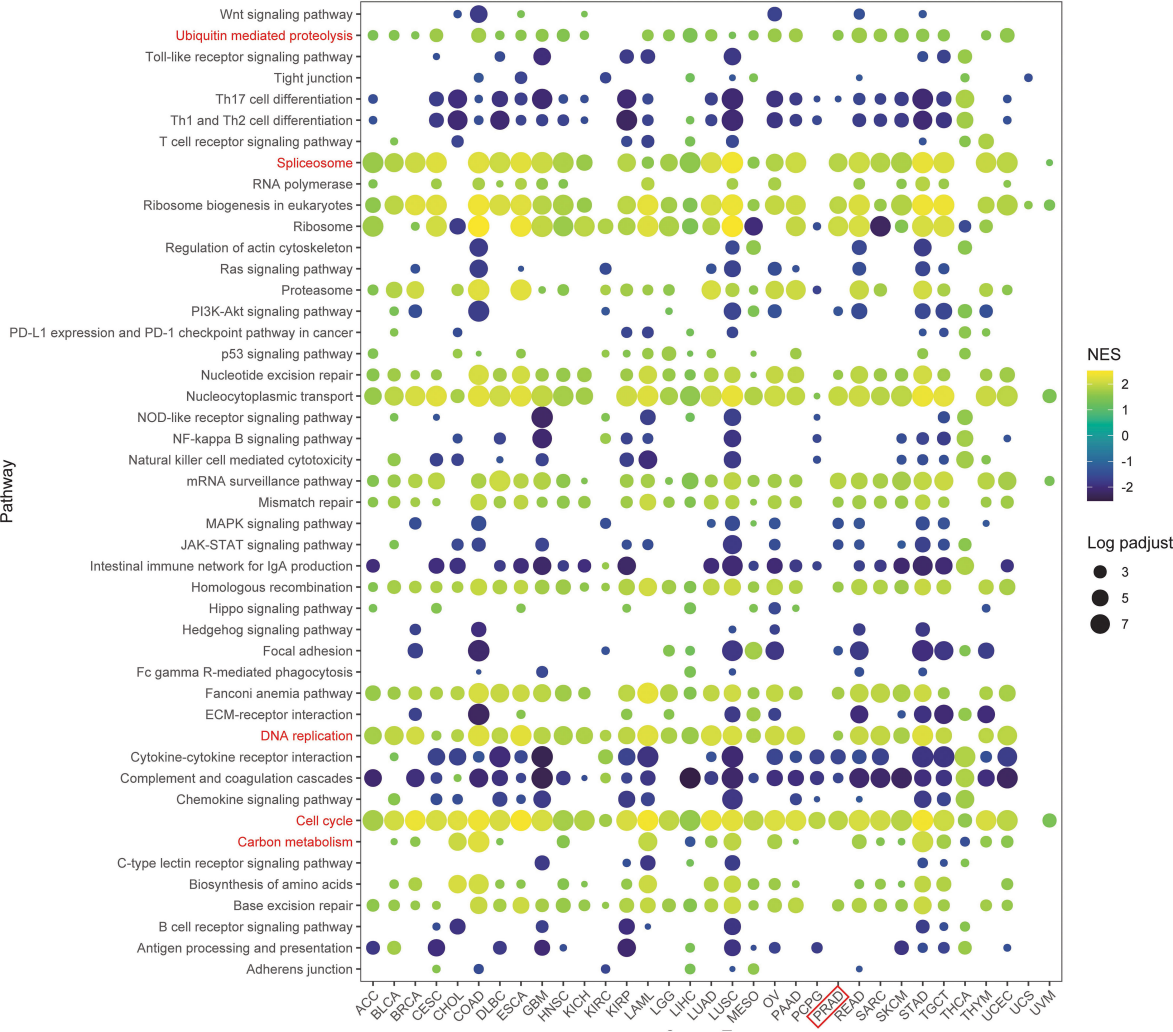
GSEA analysis of TRIP13 functions in human cancers. The bubble plot illustrates the GSEA results between TRIP13 high and TRIP13 low expression tumor patients using the KEGG gene set. Different colors indicate varying NES values, while bubble sizes reflect the corresponding log-adjusted *p*-value. Adjusted *p*-value <0.05 indicated statistical significance.

To further investigate the impact of TRIP13 on tumor patient prognosis, a pan-cancer gene set enrichment analysis (GSEA) was conducted using differentially expressed genes between the high- and low-TRIP13 expression groups. The resulting bubble plot revealed that TRIP13 activated pathways such as ubiquitin-mediated proteolysis, cell cycle, DNA repair, carbon metabolism, and spliceosome across multiple tumor types, particularly in TRIP13 high expression patients. In the TRIP13 high expression group, pathways such as Th17 cell differentiation, Th1 and Th2 cell differentiation, natural killer cell-mediated cytotoxicity, cytokine–cytokine receptor interaction, and complement and coagulation cascades were significantly inhibited. These findings suggest that TRIP13 may play a role in suppressing the antitumor immune response in these cancers (**Figure 4**). These findings align strongly with previous studies, suggesting that TRIP13 contributes to tumor progression through these pathways (21–26). Using GSVA enrichment analysis, pathway scores underwent calculation for cell cycle, DNA replication, and ubiquitin-mediated proteolysis, followed by Spearman correlation analysis. Across the comprehensive dataset, TRIP13 expression exhibited strong positive correlations with the cell cycle (*R* = 0.42, *p* < 0.001), DNA replication (*R* = 0.42, *p* < 0.001), and ubiquitin-mediated proteolysis (*R* = 0.18, *p* < 0.001). Stratified analysis by cancer type highlighted significant correlations between TRIP13 and the cell cycle in nearly all cancers, with PRAD showing a strong association (*R* > 0.64, *p* < 0.001). Additionally, notable positive correlations emerged between TRIP13 and ubiquitin-mediated proteolysis in cancers such as MESO, PRAD, and BLCA. However, in specific cancers such as PCPG, KIRC, PAAD, and CHOL, no significant correlations appeared (**Supplementary Figure 6**).

These findings illustrate the intricate relationship between elevated TRIP13 levels and increased proliferative activity, ubiquitin-mediated proteolysis, and immunosuppressive mechanisms in human cancers. The results emphasize TRIP13’s critical role in influencing key oncogenic pathways, advancing the understanding of its contributions to tumor progression and therapy resistance.

### Effect of TRIP13 expression on immune cell infiltration in human cancers

**Figure 5:**
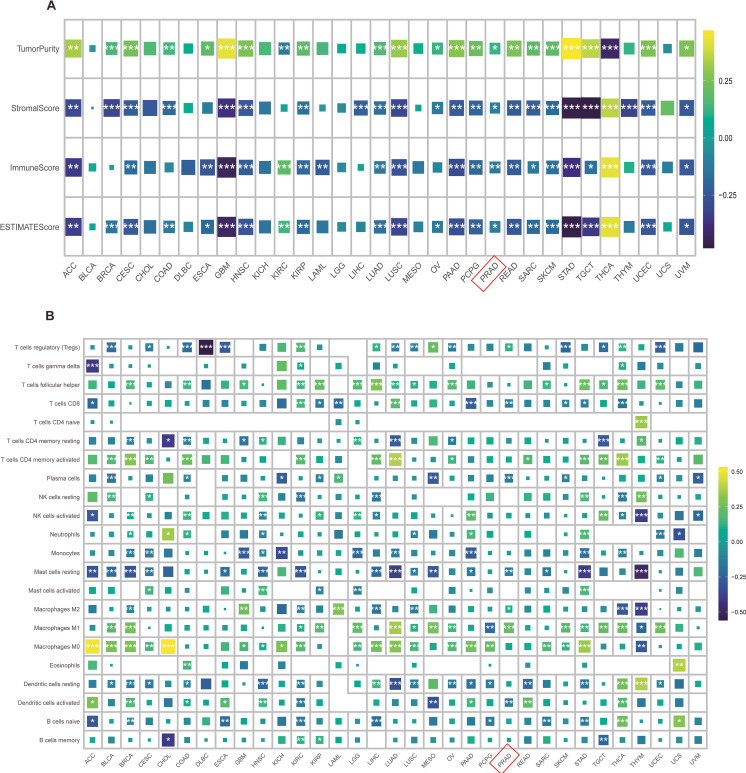
Correlation between TRIP13 expression and immune cell infiltration. **(A)** Correlation of TRIP13 with tumor purity, stromal score, immune score, and ESTIMATE score. **(B)** Correlation plot between TRIP13 expression and 22 immune cell types. Different colors represent varying correlation coefficients; * indicates *p* < 0.05; ** indicates *p* < 0.01; *** indicates *p* < 0.001; gray represents null values.

Recognizing TRIP13 as a pro-carcinogenic gene in various tumors and the intricate role of the tumor immune microenvironment in tumor development, we analyzed its association with immune cells. Correlation analyses examined TRIP13’s relationship with immune scores, stromal scores, and tumor purity. The results revealed a positive correlation between TRIP13 and tumor purity in PRAD, STAD, ACC, and GBM, while immune cell scores and stromal cell scores exhibited significant negative correlations with TRIP13. Interestingly, in THCA, the trend was completely opposite, highlighting the unique heterogeneity of the tumor microenvironment across different cancers. These findings suggest that the interaction between TRIP13 and the tumor microenvironment varies by cancer type, with THCA showing distinct patterns (**Figure 5A**). Building on these findings, we used the CIBERSORT R package to assess immune cell infiltration across cancers and analyzed the correlations between TRIP13 and various immune cell types. Interestingly, the relationship between TRIP13 and immune cells varied significantly across tumors, demonstrating notable heterogeneity. For example, TRIP13 showed a negative correlation with CD8^+^ T cells in cancers such as ACC, KIRP, LAML, PAAD, PRAD, SKCM, STAD, and THCA, but a positive correlation in KIRC and LUAD. Similarly, M1 macrophages displayed a positive correlation with TRIP13 in cancers like PRAD and a negative correlation in THYM. Conversely, M2 macrophages positively correlated with TRIP13 in PRAD but negatively in THYM. These results highlight the complexity and heterogeneity of the tumor immune microenvironment across different cancers (**Figure 5B**).

### Association of TRIP13 with immune-related regulators, TMB, and MSI

**Figure 6:**
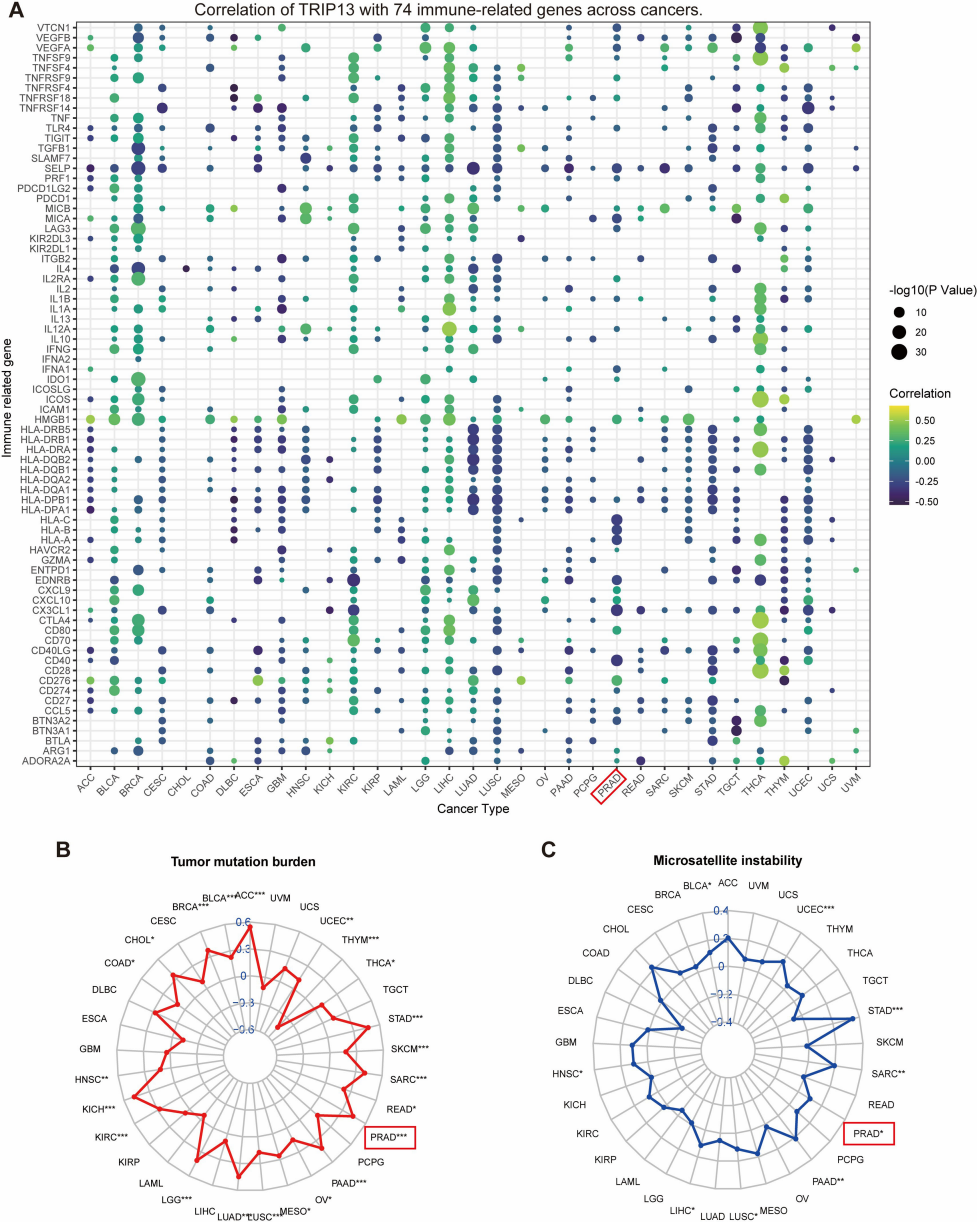
Association of TRIP13 with immune-related regulators, TMB, and MSI. **(A)** Bubble plots depict the correlation between TRIP13 and expression levels of 74 immune genes across cancer types. Colors indicate correlation coefficients, while bubble sizes represent *p*-values. **(B, C)** Radar plots showing the correlation between TRIP13 expression and tumor mutational burden (TMB, left, **B**) and microsatellite instability (MSI, right, **C**) across multiple tumor types. * indicates *p* < 0.05; ** indicates *p* < 0.01; *** indicates *p* < 0.001; gray represents null values.

Immune-related regulators play a critical role in shaping the tumor microenvironment and affecting the efficacy of cancer immunotherapy (27). The findings of our study are presented in a bubble diagram. An intriguing observation highlights the heterogeneity among tumors. For example, antigen presentation and HLA molecules display significant negative correlations with TRIP13 in tumors like LUAD and LUSC while showing positive correlations in THCA. Immunomodulatory genes exhibit predominantly positive correlations with TRIP13 in LIHC, LGG, and THCA, but tend toward negative correlations in tumors such as LUSC and GBM. In prostate cancer, the correlation between TRIP13 and various immune genes appears less consistent, showing overall negative trends and fewer significant correlations. This finding aligns with the immune-infiltrating cell correlation analysis and suggests a poorer immunotherapy response in prostate cancer (**Figure 6A**). Given the significance of TMB and MSI as key biomarkers for immunotherapy, the correlation between TMB/MSI and TRIP13 expression was analyzed across multiple cancer types. Our analysis revealed a significant positive correlation between TRIP13 and TMB in UCEC, STAD, PRAD, PAAD, and LUAD while showing a significant negative correlation in THYM (**Figure 6B**). High TRIP13 expression levels correlated positively with MSI in UCEC, STAD, SARC, PRAD, LIHC, and BLCA (**Figure 6C**). These findings suggest that TRIP13 holds potential as a predictive marker for immunotherapy response.

### Pan-cancer mutation landscape of TRIP13 and associated genes

**Figure 7:**
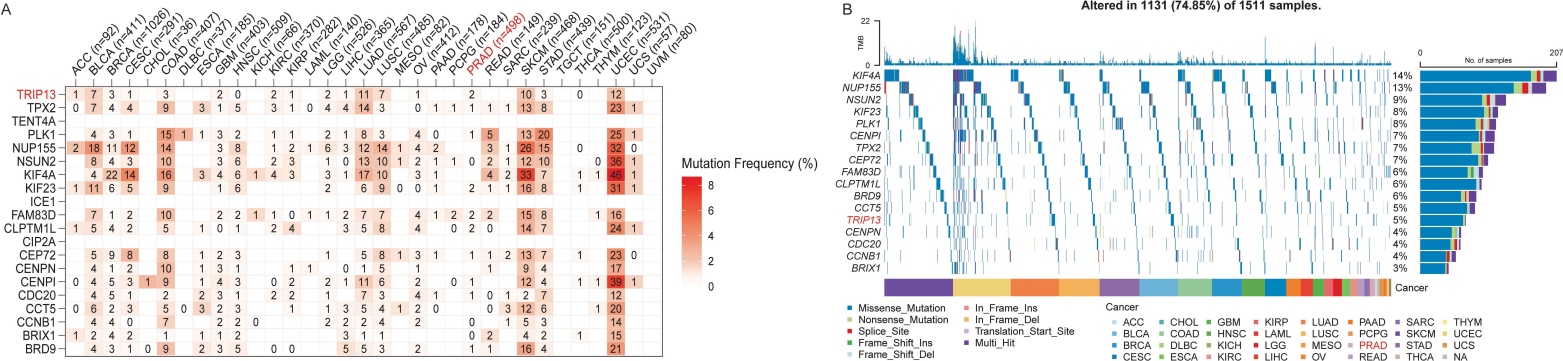
Pan-cancer mutation landscape of TRIP13 and associated genes. **(A)** The heatmap shows the mutation frequency of TRIP13 and related genes in different cancer types from the TCGA. The color gradient represents the mutation frequency. Red indicates higher mutation rates. The number inside each cell indicates the mutation frequency (%) in each cancer type. **(B)** Oncoplot visualizing the mutation profile of 20 genes across 1,511 tumor samples (1,131 samples with alterations, 74.85%). The top bar plot represents the TMB, while the side bar plot shows the number and percentage of samples with mutations for each gene. Different mutation types are color-coded. The bottom annotation bar indicates the cancer types.

TRIP13 was found to be associated with TMB and with significant activation of the cell cycle and other pathways, as revealed by GSEA enrichment analysis in multiple tumor types. Since most CCLE data represent tumor cells, we utilized the CCLE dataset to identify genes significantly and positively correlated with TRIP13 expression (Spearman correlation coefficient > 0.6). A total of 20 genes were identified, including BRD9, BRIX1, CCNB1, CCT5, CDC20, CENPI, CENPN, CEP72, CIP2A, CLPTM1L, FAM83D, ICE1, KIF23, KIF4A, NSUN2, NUP155, PLK1, TENT4A, TPX2, and TRIP13, most of which are closely linked to cell cycle regulation. To further investigate the genetic landscape, we integrated the TCGA data to analyze the mutation frequencies and distribution patterns of these genes. In the TCGA dataset, genes significantly associated with TRIP13 exhibited varying mutation frequencies across cancer types (**Figure 7A**). Notably, KIF4A, NUP155, NSUN2, and KIF23 showed high mutation frequencies in several cancer types. Oncoplot analysis highlighted the main mutation types for these genes, including missense mutations, splice site mutations, and frameshift insertions/deletions (**Figure 7B**). Among the cancer types analyzed, TRIP13 and related genes exhibited higher mutation rates in lung cancers (LUAD, LUSC) and lower mutation rates in tumors such as PCPG and PRAD. These findings suggest that TRIP13-related genes may play crucial regulatory roles in the progression of these cancers.

### TRIP13 expression and clinicopathological correlations in prostate cancer

**Figure 8:**
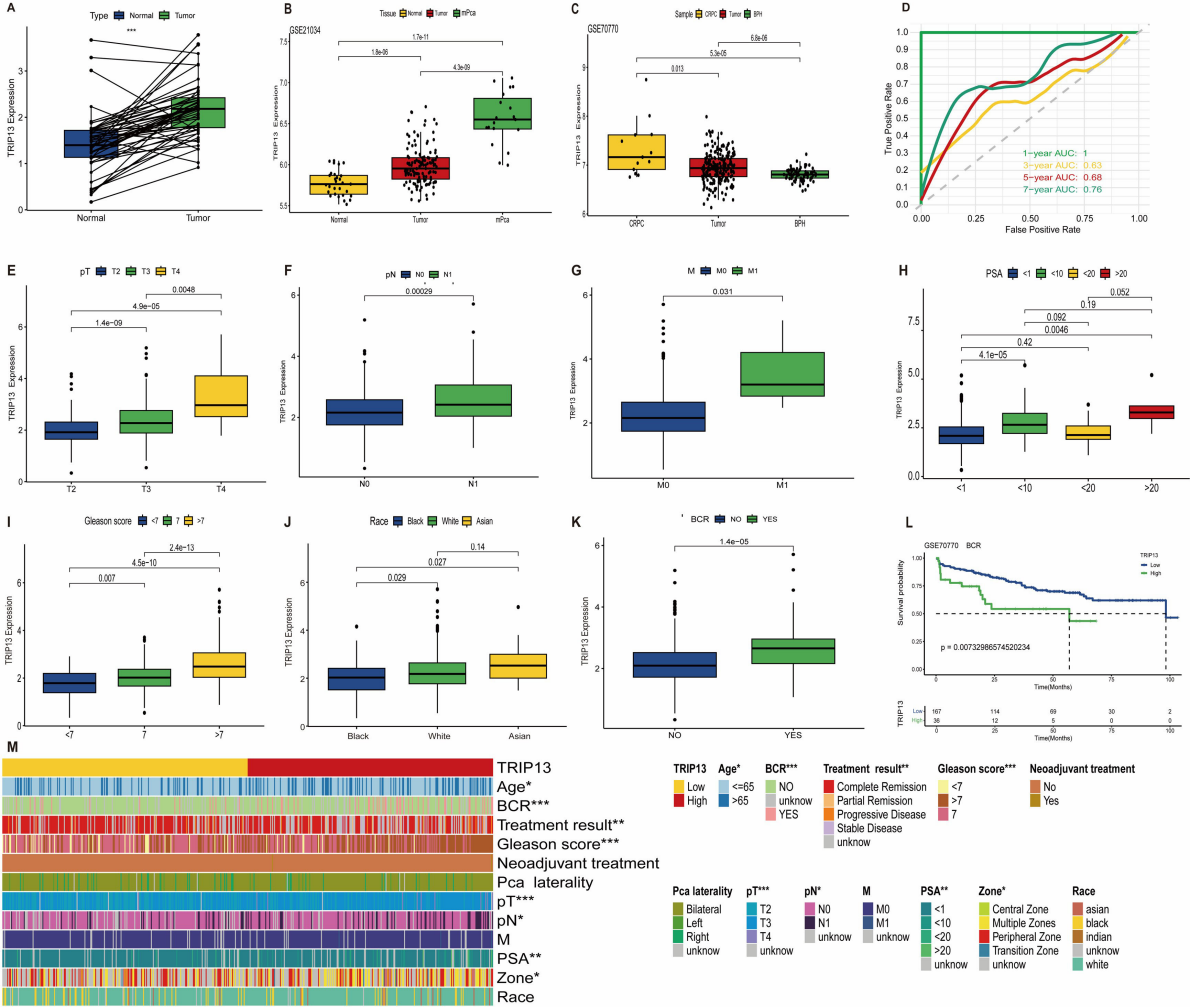
TRIP13 expression and clinicopathological correlations in prostate cancer. **(A)** Paired analysis of TRIP13 expression in normal (blue) and tumor (green) tissues in the TCGA dataset. **(B)** TRIP13 mRNA expression in the GSE21034 dataset showed significantly higher expression in tumor (red) and metastasis (green) tissues compared to normal tissues (yellow). **(C)** TRIP13 mRNA expression in the GSE70770 dataset, highlighting elevated levels in CRPC tissues (yellow) compared to tumor (red) tissues and benign prostatic hyperplasia tissues (BPH, green). **(D)** The ROC curves illustrate the predictive accuracy of TRIP13 expression for OS at 1 year (green), 3 years (yellow), 5 years (red), and 7 years (dark green). The corresponding area under the curve (AUC) values are 1.00, 0.63, 0.68, and 0.76, respectively. The dashed diagonal line represents the reference line (AUC = 0.5), indicating no predictive ability. Clinicopathologic features: **(E)** pathologic T stage. **(F)** Lymph node metastasis or not. **(G)** Distant metastasis; **(H)** prostate-specific antigen (PSA). **(I)** Gleason scores. **(J)** Race. **(K)** Biochemical recurrence (BCR). **(L)** Biochemical recurrence survival curves in GSE70770. **(M)** Heatmap showing correlations between clinicopathologic features of prostate cancer and TRIP13. * indicates *p* < 0.05; ** indicates *p* < 0.01; *** indicates *p* < 0.001.

Building on previous studies identifying TRIP13 as a risk factor in prostate cancer, we conducted further investigations into its role in the disease. In prostate cancer, TRIP13 expression levels increased significantly with higher tumor malignancy (**Figures 8A–C**). The ROC curves revealed AUC values for TRIP13 predicting survival at 1, 3, 5, and 7 years as 1.00, 0.63, 0.68, and 0.76, respectively (**Figure 8D**). These findings suggest that TRIP13’s predictive accuracy for long-term survival improves over time, indicating its potential as a robust biomarker gene. In clinical TNM staging, TRIP13 expression increased with higher disease grades (**Figures 8E–G**). Regarding PSA levels, TRIP13 expression was significantly higher in the 1–10ng/ml and >20ng/mL groups compared to the <1ng/mL group (**Figure 8H**). TRIP13 expression also rose with increasing Gleason scores (**Figure 8I**), reaching the highest levels in the Asian population (**Figure 8J**). Elevated TRIP13 expression was observed in the biochemical recurrence group, and further analysis of GSE70770 data from the GEO database confirmed that patients with high TRIP13 expression were more likely to experience biochemical recurrence (**Figures 8K, L**). Chi-square tests demonstrated correlations between TRIP13 and clinicopathologic factors, summarized in a heatmap (**Figure 8M**). The above findings were corroborated and validated using external datasets. TRIP13 demonstrated high expression in prostate cancer tissues, and its elevated levels were associated with poor patient prognosis (**Supplementary Figures 7A–L**). These findings indicate significant TRIP13 overexpression in prostate cancer tissues, correlating with poorer patient prognosis.

### Elevated TRIP13 expression in castration-resistant prostate cancer

**Figure 9:**
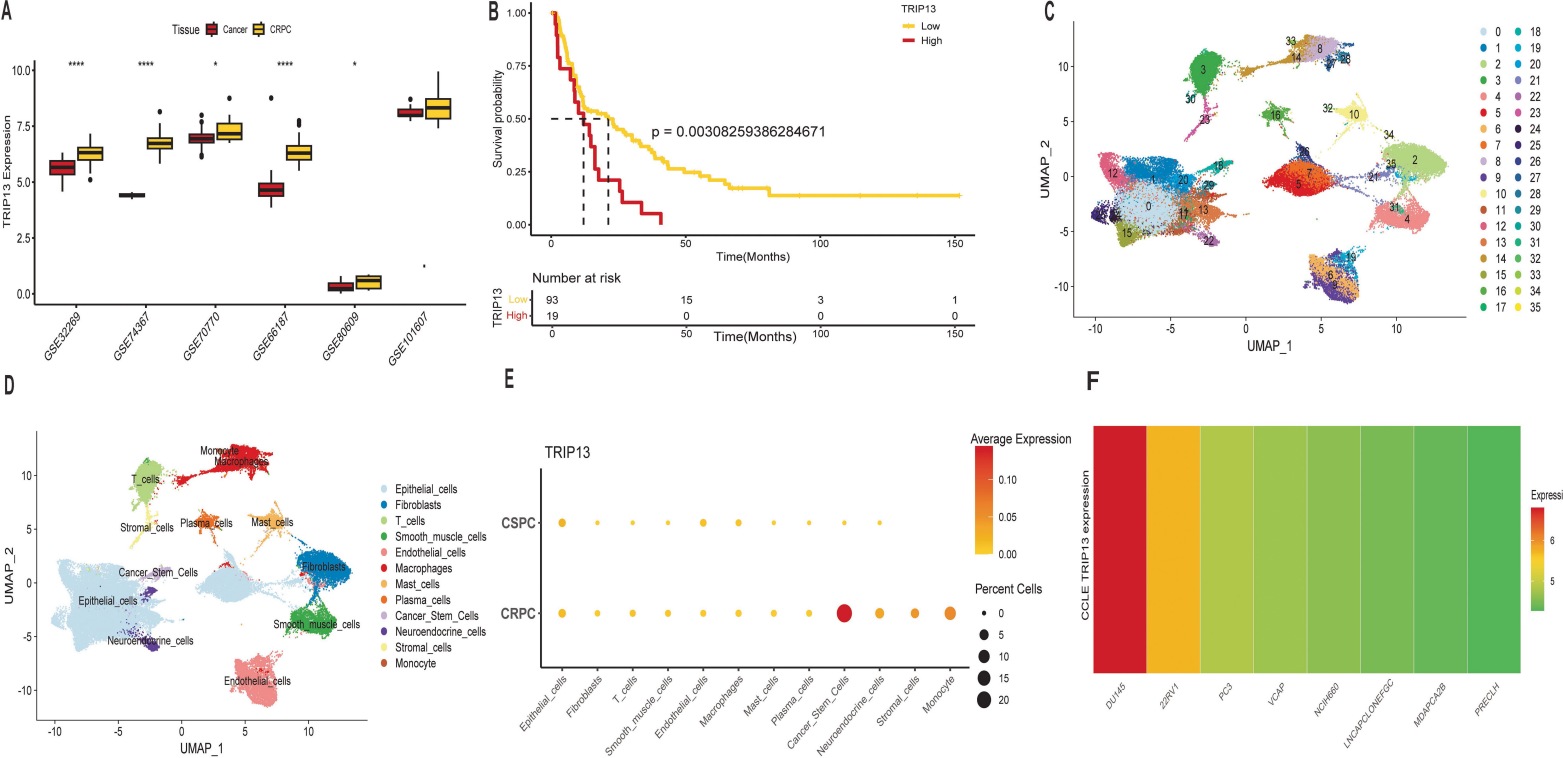
Elevated TRIP13 expression in castration resistant prostate cancer. **(A)** Boxplots displaying TRIP13 mRNA expression across tissues and datasets (GSE32269, GSE74367, GSE70770, GSE66187, GSE80609, GSE101607). * indicates p < 0.05; **** indicates p < 0.0001. **(B)** High TRIP13 expression predicts poorer prognosis in CRPC patients in GSE53922. **(C)** Uniform manifold approximation and projection (UMAP) plot showing single-cell annotation of the 36 clusters. **(D)** The UMAP plot of the 12 identified major cell types in prostate cancer. **(E)** Dot plots depicting TRIP13 mRNA expression across 12 cellular clusters. **(F)** TRIP13 mRNA expression levels in various cell lines based on CCLE data. Colors transitioning from green to red indicate TRIP13 mRNA expression levels across different cell lines.

Previous findings revealed that higher TRIP13 expression correlated with poorer prognosis and increased malignancy. Building on this, further analysis was conducted. Surprisingly, TRIP13 expression showed significant elevation in five different CRPC tissues, with an increasing trend observed in the final dataset (**Figure 9A**). In CRPC patients, high TRIP13 expression strongly correlated with poorer prognosis (**Figure 9B**). Given the significant overexpression of TRIP13 in bulk RNA-seq data and its association with poor prognosis, we analyzed single-cell data to identify the primary cell types with elevated TRIP13 expression. The UMAP plot (**Figures 9C, D**) depicts the distribution of various cell clusters. We present dot plots of manual single-cell annotations (**Supplementary Figure 8A**) alongside heatmaps of the top 10 highly expressed genes for each cell cluster (**Supplementary Figure 8B**). We identified that TRIP13 exhibits predominant overexpression in cancer stem cells (**Figure 9E**, **Supplementary Figure 8I**). Tumor stem cells typically exhibit rapid division, aligning with our earlier observation that TRIP13 promotes cell cycle activation. The tumor immune microenvironment plays a critical role in tumor development. Cancer stem cells in CRPC tissues were classified into TRIP13-positive and TRIP13-negative expression groups based on TRIP13 expression levels. Cell communication analysis revealed strong interactions between cancer stem cells and other immune cells in the TRIP13-positive expression group. These findings suggest that TRIP13 contributes to CRPC progression not only within tumor cells but also through its influence on the tumor immune microenvironment (**Supplementary Figures 8C–H**). CCLE data also revealed significantly elevated TRIP13 expression in CRPC cell lines (**Figure 9F**). Building on bioinformatics data indicating significant TRIP13 elevation in CRPC and its association with poor prognosis, we conducted *in vitro* experiments to investigate the underlying mechanism.

### TRIP13 mediates palbociclib resistance in CRPC cells

**Figure 10:**
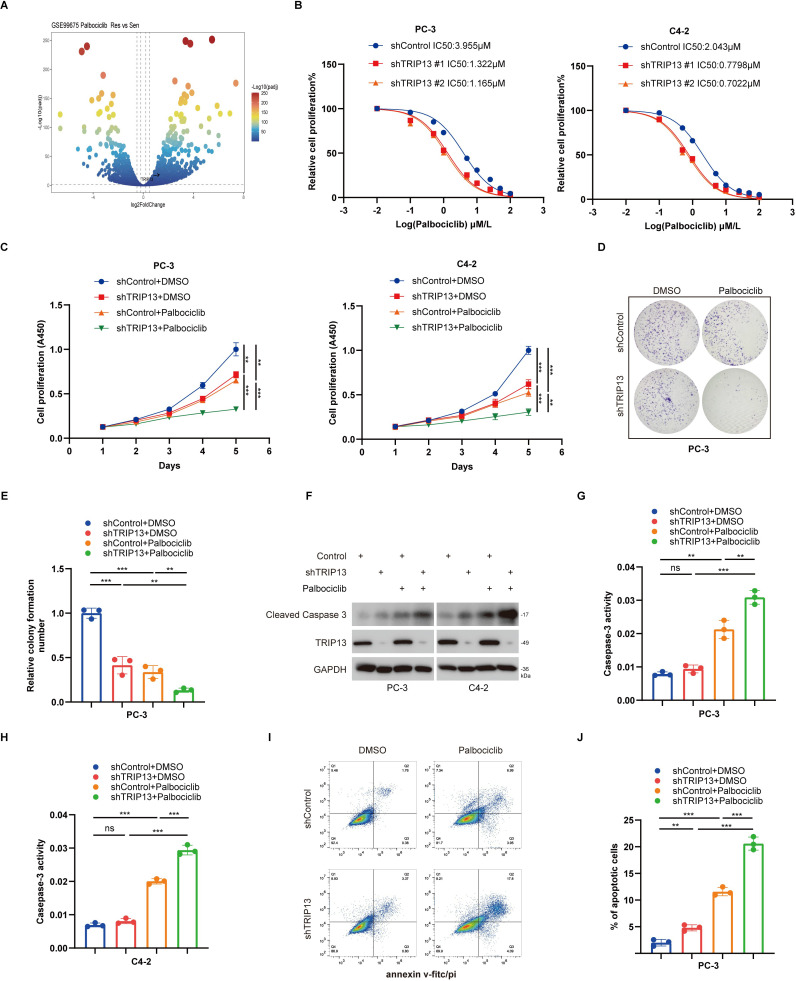
TRIP13 mediates palbociclib resistance in CRPC cells. **(A)** Volcano plot illustrating significant TRIP13 overexpression in the palbociclib-resistant group, with distinct colors indicating varying adjusted *p*-values. **(B)** PC-3 and C4–2 cells were transfected with specified shRNA for 72 h, followed by puromycin selection. The cells were then treated with incremental doses of palbociclib for 24 h and subjected to CCK-8 assays. Data were expressed as the mean ± standard error of the mean (SEM) and repeated three times. **(C)** PC-3 and C4–2 cells underwent transfection with specified shRNA for 72h. Following puromycin selection, the cells were treated with or without palbociclib (2 µM) for 24 h and subsequently collected for CCK-8 assays. Data are presented as mean ± SEM from three independent experiments. * indicates *p* < 0.05; ** indicates *p* < 0.01; *** indicates *p* < 0.001; ns, not significant. **(D, E)** PC-3 cells were transfected with specified shRNA for 72h. Following puromycin selection, the cells were treated with or without palbociclib (2 µM) for 24 h and subsequently collected for colony formation assays. Data are presented as mean ± SEM from three independent experiments. * indicates *p* < 0.05; ** indicates *p* < 0.01; *** indicates *p* < 0.001; ns, not significant. **(F–J)** PC-3 and C4–2 cells were transfected with designated shRNA for 72h. After puromycin selection, the cells were treated with or without palbociclib (2 µM) for 24 h and subsequently collected for Western blot analysis, caspase-3 activity assays, and Annexin V FITC/PI assays. Data represent the mean ± SEM from three independent biological replicates. * indicates *p* < 0.05; ** indicates *p* < 0.01; *** indicates *p* < 0.001; ns, not significant. Statistical significance was determined using the indicated tests, with *p* < 0.05 considered significant.

Given the limited therapeutic options for advanced prostate cancer and the high expression of TRIP13 in tumor stem-like cell populations within CRPC, along with the limited therapeutic benefit of CDK4/6 inhibitors in prostate cancer, we speculated that TRIP13 may be closely associated with resistance to CDK4/6 inhibitors. Based on this hypothesis, and considering our team’s long-term focus on the mechanisms of CDK4/6 inhibitors (28), we subsequently performed a systematic analysis to verify the potential role of TRIP13 in the development of resistance in prostate cancer. Additionally, analysis of the GSE99675 dataset revealed elevated TRIP13 expression in the resistance group (**Figure 10A**). Based on these findings, we conducted further *in vitro* experiments to investigate this relationship. To explore whether TRIP13 contributes to palbociclib resistance in CRPC cells, we evaluated its role in modulating drug sensitivity. CRPC cell lines were transfected with TRIP13-specific shRNA for knockdown or TRIP13-labeled plasmids for overexpression. Our findings revealed that silencing TRIP13 significantly decreased the median inhibitory concentration (IC50) of palbociclib (**Figure 10B**), suggesting a role for TRIP13 in mediating palbociclib resistance. CCK-8 and colony formation assays demonstrated that TRIP13 knockdown increased the sensitivity of CRPC cells to palbociclib (**Figures 10C–E**). Additionally, TRIP13 knockdown enhanced apoptosis following palbociclib treatment (**Figures 10F–J**). Overexpression of TRIP13 promoted resistance to palbociclib, as demonstrated in **Supplementary Figures 9A–I**. Together, these findings highlight TRIP13 as a key regulator of prostate cancer cell sensitivity to palbociclib therapy.

### E2F1 transcriptionally activates TRIP13 to drive palbociclib resistance in prostate cancer

**Figure 11:**
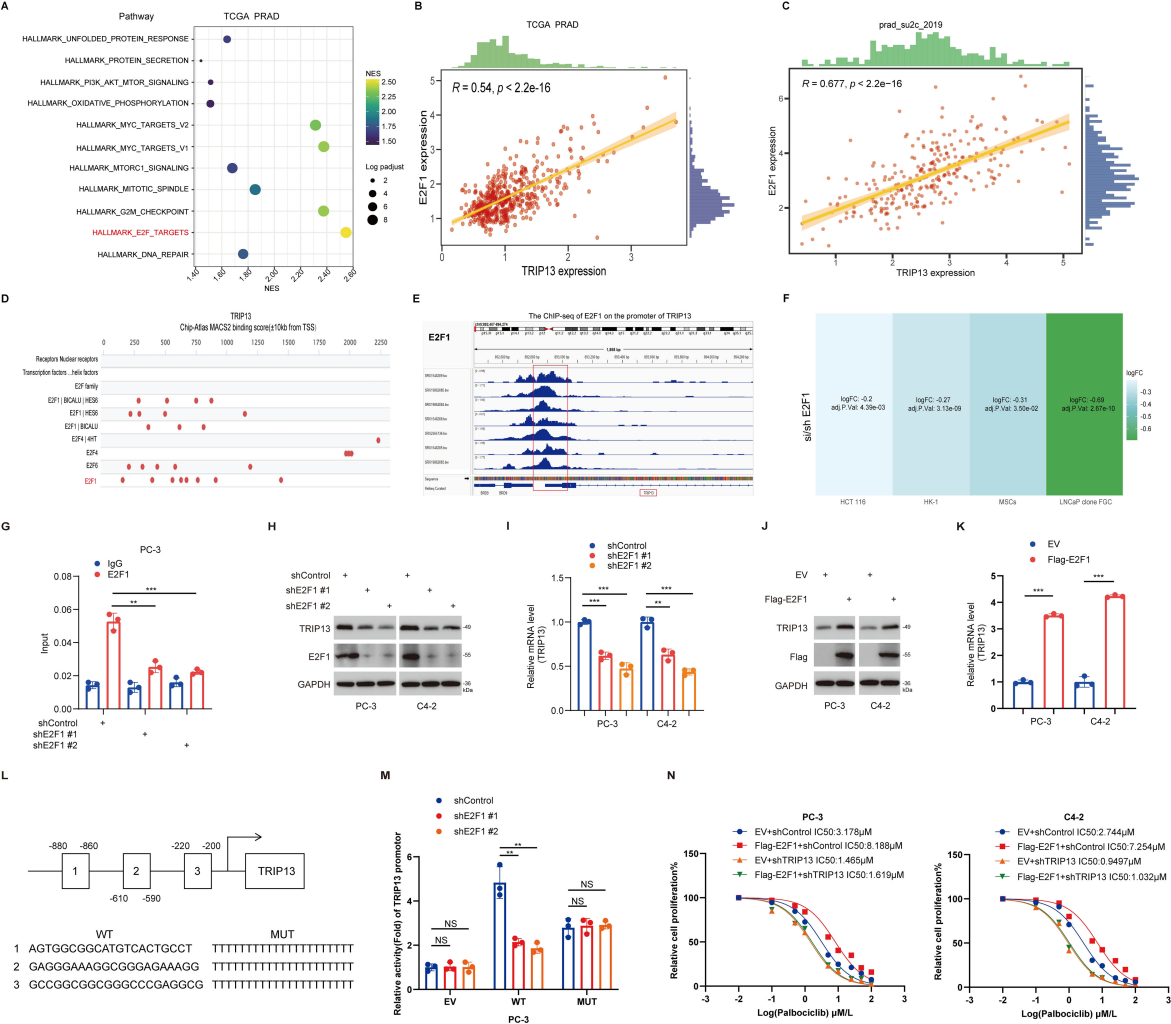
E2F1 transcriptionally activates TRIP13 to drive Palbociclib resistance in prostate cancer. In TCGA prostate cancer, samples were stratified into high and low TRIP13 expression groups. Differential expression analysis was performed, followed by GSEA enrichment analysis. In the bubble plot, different colors represent NES values, while bubble sizes correspond to the log-adjusted *P*-values **(A)**. Spearman correlation plots showing the relationship between E2F1 and TRIP13, panels: **(B)** TCGA prostate cancer data, **(C)** PRAD_SU2C_2019 data. **(D)** Ominer network tool prediction of potential transcription factors for TRIP13. **(E)** Chip-Seq analysis of E2F1 binding to the TRIP13 promoter region from Chip-Atlas. **(F)** GPSAdb data indicate reduced TRIP13 expression following E2F1 knockdown. Different colors represent different LogFC. **(G)** PC-3 cells were transfected with specified shRNA for 72 hours, followed by puromycin selection. These cells were treated with collected for ChIP-qPCR analysis using IgG or E2F1 antibodies. Data represent the mean ± SEM from three independent experiments. ** indicates *P* < 0.01, *** indicates *P* < 0.001. **(H, I)** PC-3 and C4-2 cells were transfected with specified shRNA for 72 hours. After puromycin screening, cells were subjected to western blot analysis **(H)** and RT-qPCR assay **(I)**. Data represent the mean ± SEM from three independent experiments. ** indicates *P* < 0.01, *** indicates *P* < 0.001. **(J, K)** PC-3 and C4-2 cells were transfected with designated plasmids for 24 hours. Following puromycin screening, cells underwent western blot analysis (J) and RT-qPCR assay **(K)**. Data are presented as the mean ± SEM from three independent experiments. *** indicates *P* < 0.001. **(L)** The sequence and location of the E2F1 binding peak in the TRIP13 promoter. WT, wild type, MUT, mutant. **(M)** PC-3 cells were transfected with indicated plasmid for 24 h. After puromycin selection, the cells were harvested and the activity of the TRIP13 promoters was measured. Data are presented as the mean ± SEM from three independent experiments. ** indicates *P* < 0.01, ns, not significant. **(N)** PC-3 and C4-2 cells were transfected with specified shRNA for 72 hours and indicated plasmid for 24 h, followed by puromycin selection. The cells were then treated with incremental doses of palbociclib for 24 hours and subjected to CCK-8 assays. Data were expressed as the mean ± SEM and repeated three times. Statistical significance was determined using the indicated tests, with P <0.05 considered significant.

TRIP13 mediates resistance to CDK4/6 inhibitors in prostate cancer, which prompted us to further investigate the upstream transcription factors that regulate TRIP13. Based on TCGA prostate cancer data and using the HALLMARK gene sets from the msigdbr R package for GSEA enrichment analysis, we found that TRIP13 significantly activates the E2F signaling pathway (**Figure 11A**). Notably, E2F1, a key component of the E2F pathway, has been reported to play an important role in prostate cancer (28–31). Therefore, we hypothesized a potential association between E2F1 and TRIP13 and conducted additional analyses. Correlation analysis of TRIP13 and E2F1 in the PRAD_SU2C_2019 and TCGA datasets revealed a strong positive association between the two genes (**Figures 11B, C**). In addition, we used the Signaling Pathways Project (http://signalingpathways.org/index.jsf) and ChIP-Atlas (https://chip-atlas.org/) online tools to predict potential transcription factors. The results showed that E2F1, identified across both resources, may bind to the promoter region of TRIP13 (**Figures 11D, E**). Further analysis using the GPSAdb database (https://www.gpsadb.com/) demonstrated that E2F1 knockdown reduces TRIP13 mRNA expression in HCT116, HK-1, MSCs, and LNCaP clone FGC cells (**Figure 11F**). ChIP-seq results showed that E2F1 specifically binds to the promoter region of TRIP13 in PC-3 cells (**Figure 11G**). Knockdown of E2F1 reduced TRIP13 protein and mRNA levels in PC-3 and C4–2 cells (**Figures 11H, I**). Conversely, overexpression of E2F1 increased TRIP13 expression at both the protein and mRNA levels (**Figures 11J, K**). Dual-luciferase assays demonstrated that E2F1 knockdown decreased the activity of the wild-type TRIP13 promoter, but not a mutant promoter, in PC-3 cells (**Figures 11L, M**). Moreover, depletion of TRIP13 abrogated the increase in palbociclib IC50 conferred by E2F1 overexpression in prostate cancer cells (**Figure 11N**). In summary, our results demonstrate that the E2F1–TRIP13 axis drives palbociclib resistance in prostate cancer.

### TRIP13 mediates palbociclib resistance in prostate cancer by activating the ubiquitination pathway

**Figure 12:**
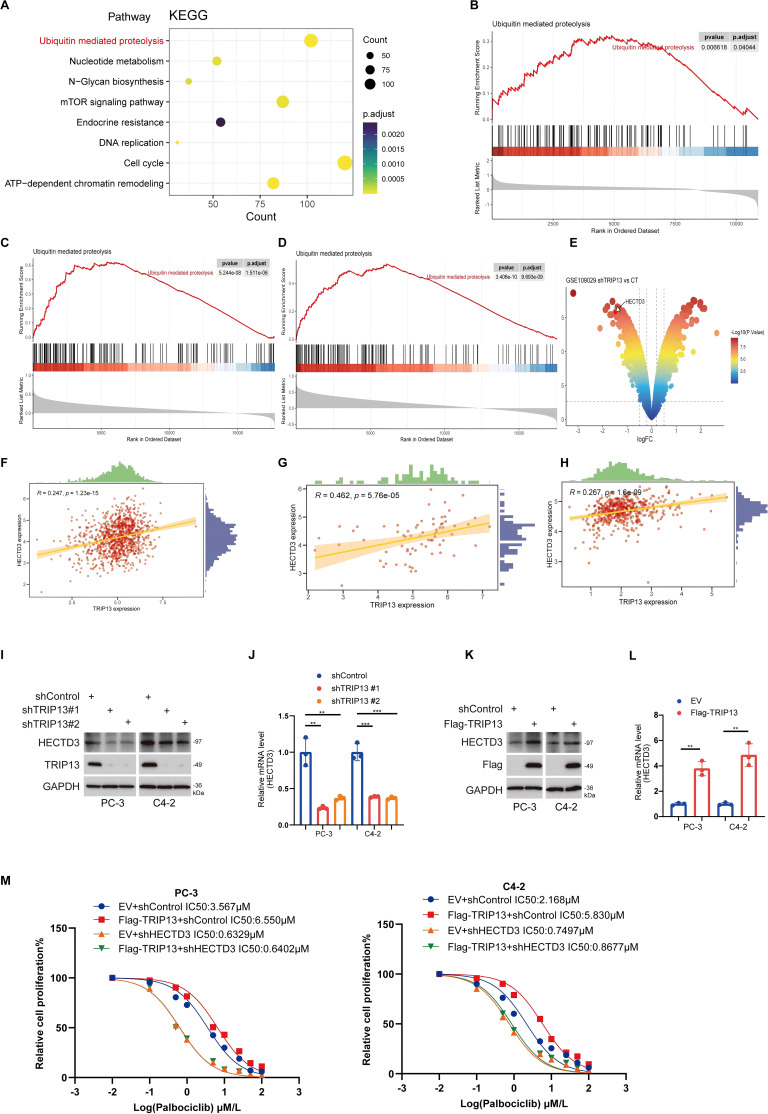
TRIP13 mediates Palbociclib resistance in prostate cancer by activating the ubiquitination pathway. **(A)** Patient specimens were categorized into TRIP13 high and low expression groups based on median expression, and highly expressed genes in the TCGA prostate cancer dataset were analyzed for KEGG enrichment. **(B)** GSEA analysis of the TCGA prostate cancer dataset after categorizing patient specimens into TRIP13 high and low expression groups. **(C, D)** GSEA enrichment analysis following correlation analysis of each gene with TRIP13 in CCLE pan-cancer data **(C)** and urologic data set **(D)**. Volcano plots of differentially expressed genes between shTRIP13 and Control Groups in GSE109029 data **(E)**. **(F-H)** HECTD3 shows a significant positive association with TRIP13 in CCLE and TCGA datasets, including CCLE pan-cancer data **(F)**, CCLE urologic data set **(G)**, and TCGA-PRAD **(H)**. **(I, J)** PC-3 and C4-2 cells were infected with indicated shRNA for 72h. Cells were harvested for the western blot analysis **(I)** and RT-qPCR analysis **(J)**. Data present as mean±SEM with three replicates. ** indicates *P* < 0.01; *** indicates *P* < 0.001. **(K, L)** PC-3 and C4-2 cells were transfected with indicated plasmids for 24h. Cells were harvested for the western blot analysis **(K)** and RT-qPCR analysis **(L)**. Data present as mean±SEM with three replicates. ** indicates *P* < 0.01. **(M)** PC-3 and C4-2 cells were transfected with specified shRNA for 72 hours and indicated plasmid for 24 h, followed by puromycin selection. The cells were then treated with incremental doses of palbociclib for 24 hours and subjected to CCK-8 assays. Data were expressed as the mean ± SEM and repeated three times. Statistical significance was determined using the indicated tests, with P <0.05 considered significant.

Our findings indicate that E2F1 promotes resistance to palbociclib in prostate cancer by transcriptionally upregulating TRIP13. To further explore the mechanisms underlying TRIP13’s role in palbociclib resistance in CRPC, we divided prostate cancer patients in the TCGA-prostate cancer dataset into high- and low-TRIP13 expression groups. KEGG (**Figure 12A**) enrichment analyses of genes highly expressed in the high-TRIP13 group revealed their involvement in the ubiquitination pathway. Subsequent GSEA enrichment analysis confirmed that TRIP13 significantly activates the ubiquitination pathway in both CCLE and TCGA-prostate cancer datasets (**Figures 12B–D**). This aligns with previous studies that linked TRIP13 to ubiquitination modifications, further supported by our observation of TRIP13-mediated activation of the ubiquitination pathway in TCGA pan-cancer GSEA analysis (**Figure 4**). We hypothesize that TRIP13 promotes CRPC palbociclib resistance by regulating ubiquitinating enzymes. Analysis of the GSE109029 bladder cancer dataset revealed that knockdown of TRIP13 led to a significant reduction in HECTD3 expression in bladder cancer (**Figure 12E**). According to current studies, HECTD3 enhances radiotherapy resistance in triple-negative breast cancer by modulating p62-dependent autophagy and DNA damage repair (32). In ovarian cancer, HECTD3 is upregulated via the HER2/STAT3 axis and contributes to platinum-based chemotherapy resistance (33). Although HECTD3 has been identified as a key regulator of cell survival under treatment stress through these core pathways, it remains unclear whether it also mediates resistance to CDK4/6 inhibitors. Considering tumor heterogeneity, we assessed the relationship between TRIP13 and HECTD3 in CCLE and TCGA datasets, identifying a strong positive correlation between the two genes (**Figures 12F–H**). These findings suggest that TRIP13 may mediate palbociclib resistance by regulating HECTD3 expression. To confirm our findings, we knocked down TRIP13, followed by RT-qPCR and Western blot experiments. We observed that TRIP13 knockdown led to a decrease in HECTD3 expression at both the protein and mRNA levels (**Figures 12I, J**). In contrast, TRIP13 overexpression resulted in an increase in HECTD3 expression at both the protein and RNA levels (**Figures 12K, L**). Notably, protein interaction studies yielded a critical finding: co−immunoprecipitation failed to detect a direct physical interaction between TRIP13 and HECTD3. The results strongly suggest that TRIP13 regulates HECTD3 not through stable protein−protein binding, but more likely at the transcriptional or post−transcriptional (mRNA) level. This points to a more complex regulatory network, in which TRIP13 may indirectly modulate HECTD3 transcription or mRNA stability through intermediate factors or signaling pathways (**Supplementary Figure 10A**). Furthermore, HECTD3 depletion prevented the TRIP13 overexpression-induced increase in palbociclib IC50 in prostate cancer cells (**Figure 12M**). Taken together, these results demonstrate that TRIP13 promotes palbociclib resistance through HECTD3 activation.

## Discussion

Cancer poses a significant threat to human life. Despite advancements in science and technology improving tumor diagnosis and treatment, the 5-year overall survival rate for many cancers remains critically low (34). This highlights the pressing need to identify new biomarkers for improved diagnosis and therapy. Previous studies have highlighted the critical role of TRIP13 in tumors (9, 11, 12, 25, 26). However, to our knowledge, no comprehensive pan-cancer analysis of TRIP13 has been conducted. In this study, we performed such an analysis and found that TRIP13 was significantly overexpressed in most tumors, with high expression linked to poor patient prognosis. This indicates that TRIP13 may act as an oncogenic factor in cancer.

In this study, we observed that TRIP13 expression increases in a broad range of tumor types, and higher TRIP13 levels are associated with poorer survival in many cancers, a pattern that supports its potential pro-tumorigenic relevance. Notably, pan-cancer survival analyses also indicate a context-dependent association in which TRIP13 correlates with improved prognosis in a subset of tumor types, such as ovarian cancer and stomach adenocarcinoma. Although this observation does not constitute the main focus of our experimental validation, it warrants a brief discussion given the well-recognized heterogeneity of pan-cancer survival associations. The prognostic impact of TRIP13 appears strongly context dependent. In highly proliferative and genomically unstable tumors, TRIP13 upregulation may facilitate cell cycle progression and treatment resistance, leading to poor outcomes. In contrast, in certain cancer types or molecular subgroups, elevated TRIP13 expression may associate with distinct lineage states or therapeutic responsiveness, resulting in improved prognosis. Thus, the clinical relevance of TRIP13 depends more on a tumor context than on expression level alone.

To investigate its role in driving cancer progression, we conducted GSEA enrichment analysis to uncover potential underlying mechanisms. GSEA enrichment revealed that TRIP13 overexpression in many tumors significantly activated pathways such as the cell cycle, ubiquitination, and DNA repair. Recent literature highlights that TRIP13 is implicated in ubiquitination modification, cell cycle regulation, and DNA repair across various cancers, including gastric cancer (21), breast cancer (23), pancreatic ductal adenocarcinoma (35), lung adenocarcinoma (36), and head and neck squamous cell carcinoma (HNSC) (11), respectively. These findings support the reliability of our bioinformatics data analysis. Our study indicates that TRIP13 mediates ubiquitination modification in CRPC by upregulating HECTD3 expression. Literature reports further revealed that HECTD3 facilitates the malignant proliferation of gastric cancer through K29-linked polyubiquitination of c-MYC (37). We speculate that HECTD3 contributes to CRPC progression or resistance by mediating the ubiquitination of target proteins, warranting further investigation in future studies. This study is the first to demonstrate that TRIP13 overexpression reduces the sensitivity of prostate cancer to CDK4/6 inhibitors. We confirmed that E2F1 functions as a transcription factor for TRIP13 and promotes its expression. Our team previously found that CBX3 binds to RB1 to release E2F1, thereby accelerating cell cycle progression and decreasing the sensitivity of CRPC to CDK4/6 inhibitors (31). Based on our results, we speculate that E2F1 enhances TRIP13 expression and subsequently drives the upregulation of HECTD3, thereby contributing to CDK4/6 inhibitor resistance.

Additionally, the relationship between TRIP13 and immune cells within tumor microenvironments exhibited significant heterogeneity. Prostate cancer immunotherapy remains largely ineffective, and at the single-cell level, we identified strong interactions between TRIP13-positive expression tumor cells and various immune cells. This finding suggests that TRIP13-driven tumor progression may be linked to the tumor immune microenvironment. Furthermore, TRIP13 showed a negative correlation with CD274 (*R* = −0.122, *p* = 0.0063) and CD8 in T cells (*R* = −0.136, *p* = 0.0024) while displaying a positive correlation with M1 macrophages (*R* = 0.232, *p* = 1.86e−07) in PRAD. These results highlight the complexity of the tumor immune microenvironment, indicating that TRIP13 may act as a regulator in this process, which warrants further investigation.

In summary, this study provides novel insights into TRIP13 as a driver of tumor progression and therapy resistance, particularly in prostate cancer, emphasizing its potential as a biomarker and therapeutic target. However, our study has certain limitations, as it remains unclear whether TRIP13 contributes to tumor progression through other pathways. Future investigations with comprehensive experimental validation are needed to clarify the precise mechanisms of TRIP13-mediated tumorigenesis and drug resistance, thereby deepening our understanding of its role and potential as a therapeutic target in cancer.

## Conclusions

In summary, our study shows that TRIP13 exhibits marked upregulation in most cancer tissues compared with normal counterparts and functions as a key driver of tumor biology by promoting cell proliferation, participating in ubiquitination processes, and enhancing DNA repair. In prostate cancer, high TRIP13 expression drives resistance to CDK4/6 inhibitors. Mechanistically, we delineate an E2F1–TRIP13–HECTD3 axis, in which E2F1 transcriptionally upregulates TRIP13, and TRIP13 in turn elevates HECTD3, thereby further reinforcing resistance to CDK4/6 inhibition.

## Data Availability

The datasets presented in this study can be found in online repositories. The names of the repository/repositories and accession number(s) can be found in the article/**Supplementary Material**.

## Glossary

ACC: adrenocortical carcinoma
BLCA: bladder cancer
BRCA: breast cancer
CESC: cervical squamous cell carcinoma and endocervical adenocarcinoma
CHOL: cholangiocarcinoma
COAD: colon adenocarcinoma
DLBC: diffuse large B-cell lymphoma
ESCA: esophageal cancer
GBM: glioblastoma multiforme
HNSC: head and neck squamous cell carcinoma
KICH: kidney chromophobe
KIRC: kidney renal clear cell carcinoma
KIRP: kidney renal papillary cell carcinoma
LAML: acute myeloid leukemia
LGG: lower grade glioma
LIHC: liver cancer
LUAD: lung adenocarcinoma
LUSC: lung squamous cell carcinoma
MESO: mesothelioma
OV: ovarian cancer
PAAD: pancreatic cancer
PCPG: pheochromocytoma and paraganglioma
PRAD: prostate cancer
READ: rectal cancer
SARC: sarcoma
SKCM: skin cutaneous melanoma
STAD: stomach cancer
TGCT: testicular germ cell tumors
THCA: thyroid cancer
THYM: thymoma
UCEC: uterine corpus endometrial carcinoma
UCS: uterine carcinosarcoma
UVM: uveal melanoma
HR: hazard ratio
TMB: tumor mutational burden
MSI: microsatellite instability
TCGA: The Cancer Genome Atlas
GEO: Gene Expression Omnibus
GO: Gene Ontology
KEGG: Kyoto Encyclopedia of Genes and Genomes
GSEA: gene set enrichment analysis
GSVA: gene set variation analysis
K-M: Kaplan–Meier
DEG: differentially expressed genes
OS: overall survival
DSS: disease-specific survival
DFS: disease-free survival
PFS: progression-free survival
UMAP: uniform manifold approximation and projection
PSA: prostate-specific antigen
BCR: biochemical recurrence.
