# Supplementary material for: Multi-omics analysis and functional validation reveal the oncogenic role of TRIP13
Source: Front Immunol. 2026 Apr 22;17:1691436. doi: 10.3389/fimmu.2026.1691436 (PMC13143902; doi:10.3389/fimmu.2026.1691436)
Supplement: Supplementary file 2 [file DataSheet2.pdf]

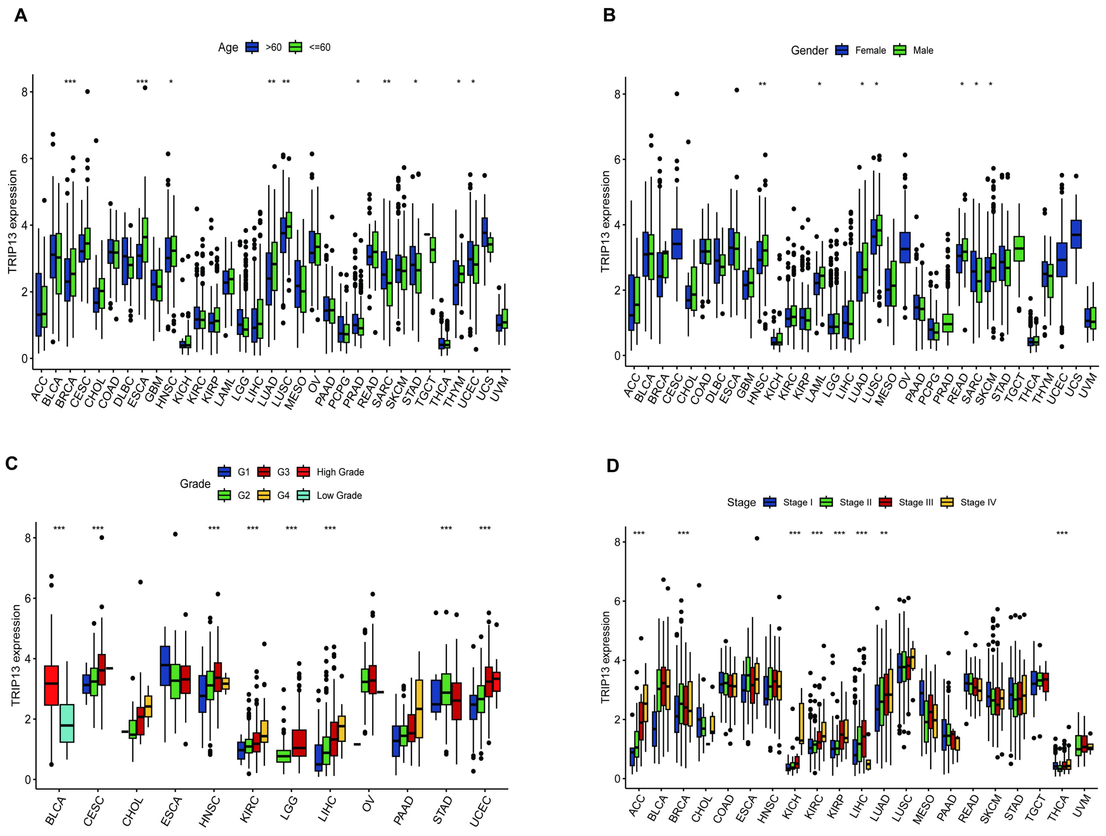

Supplementary figure 1. Differential *TRIP13* expression across pan-cancer clinicopathologic subgroups. (A) Age based groups, (B) Gender based groups, (C)Grade based groups, (D) Stage based groups. * Indicates p < 0.05, ** indicates p < 0.01, *** indicates p < 0.001, and grey represents null values.

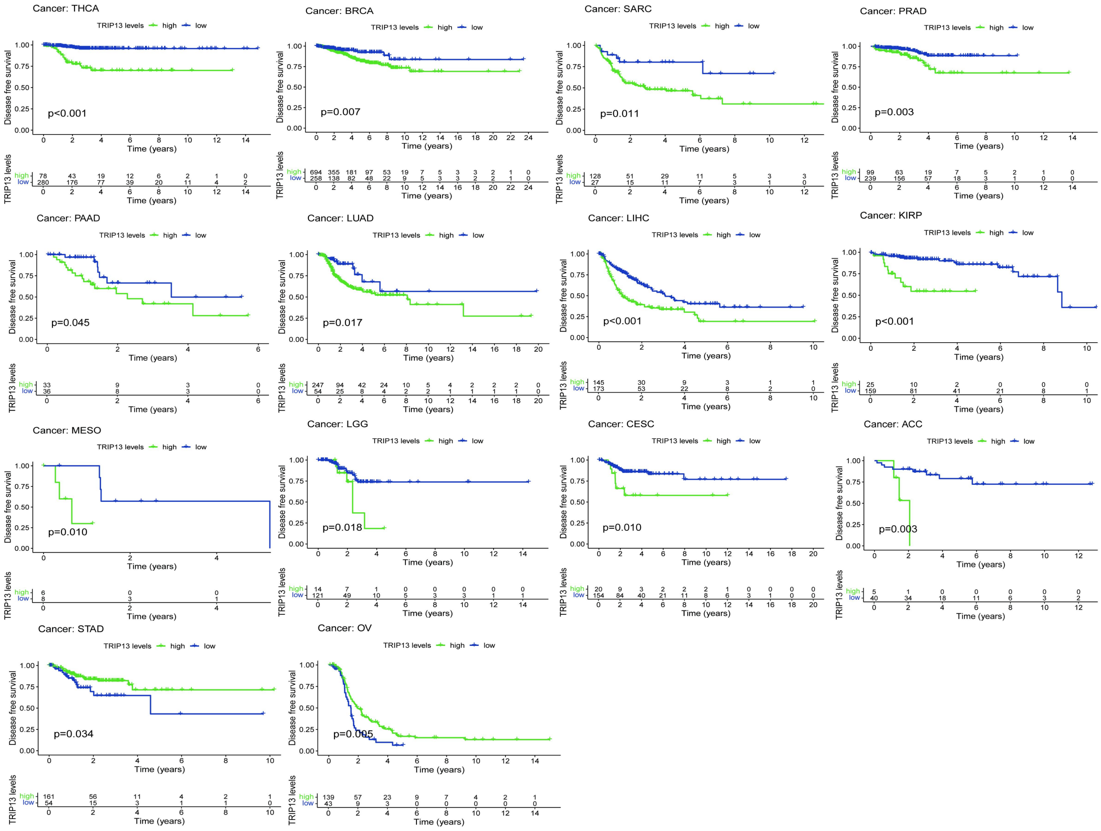

Supplementary figure 2. Predictive value of *TRIP13* expression in disease free survival (DFS) in pan-cancer.

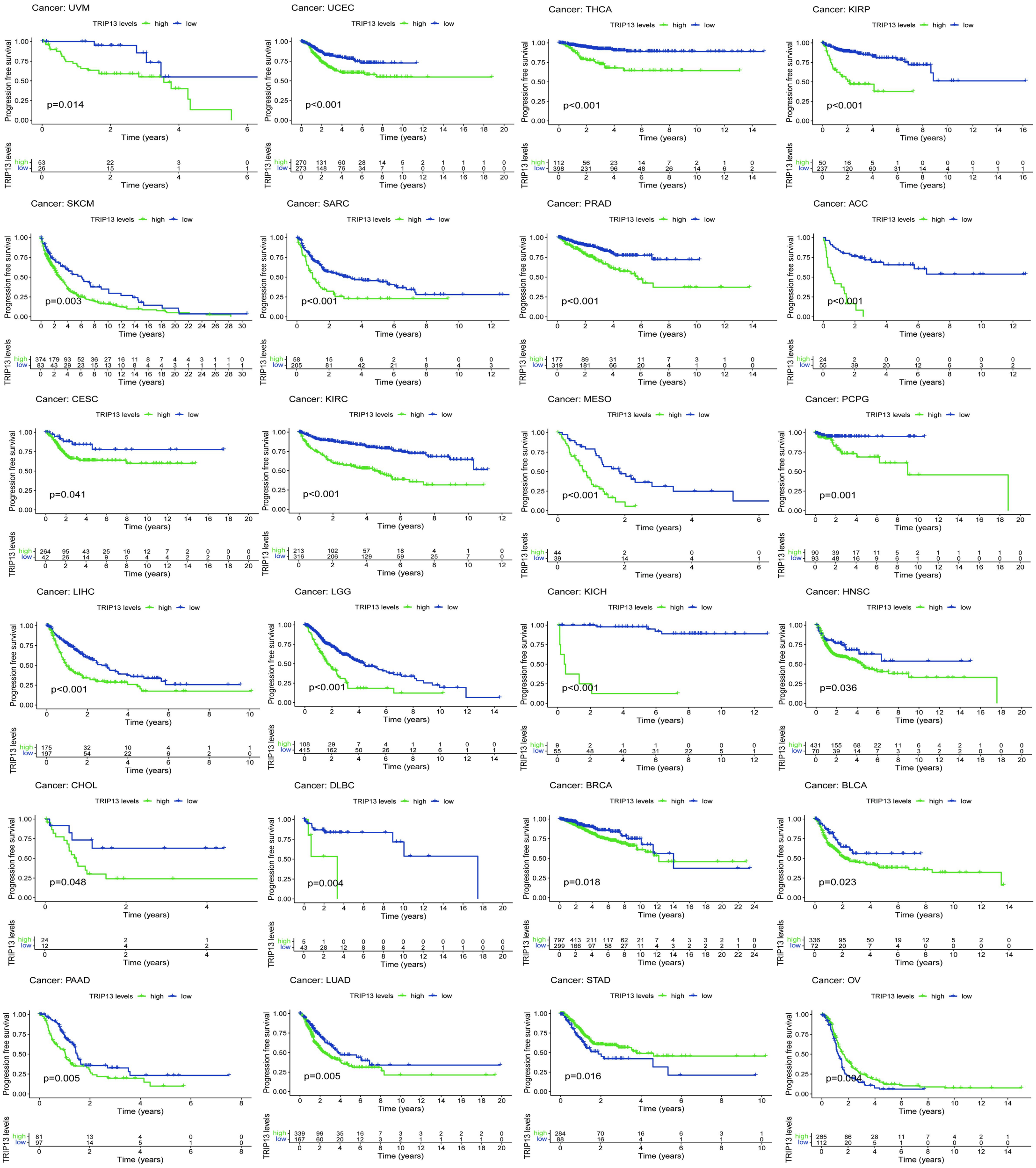

Supplementary figure 3. Predictive value of *TRIP13* expression in progression free survival (PFS) in pan-cancer.

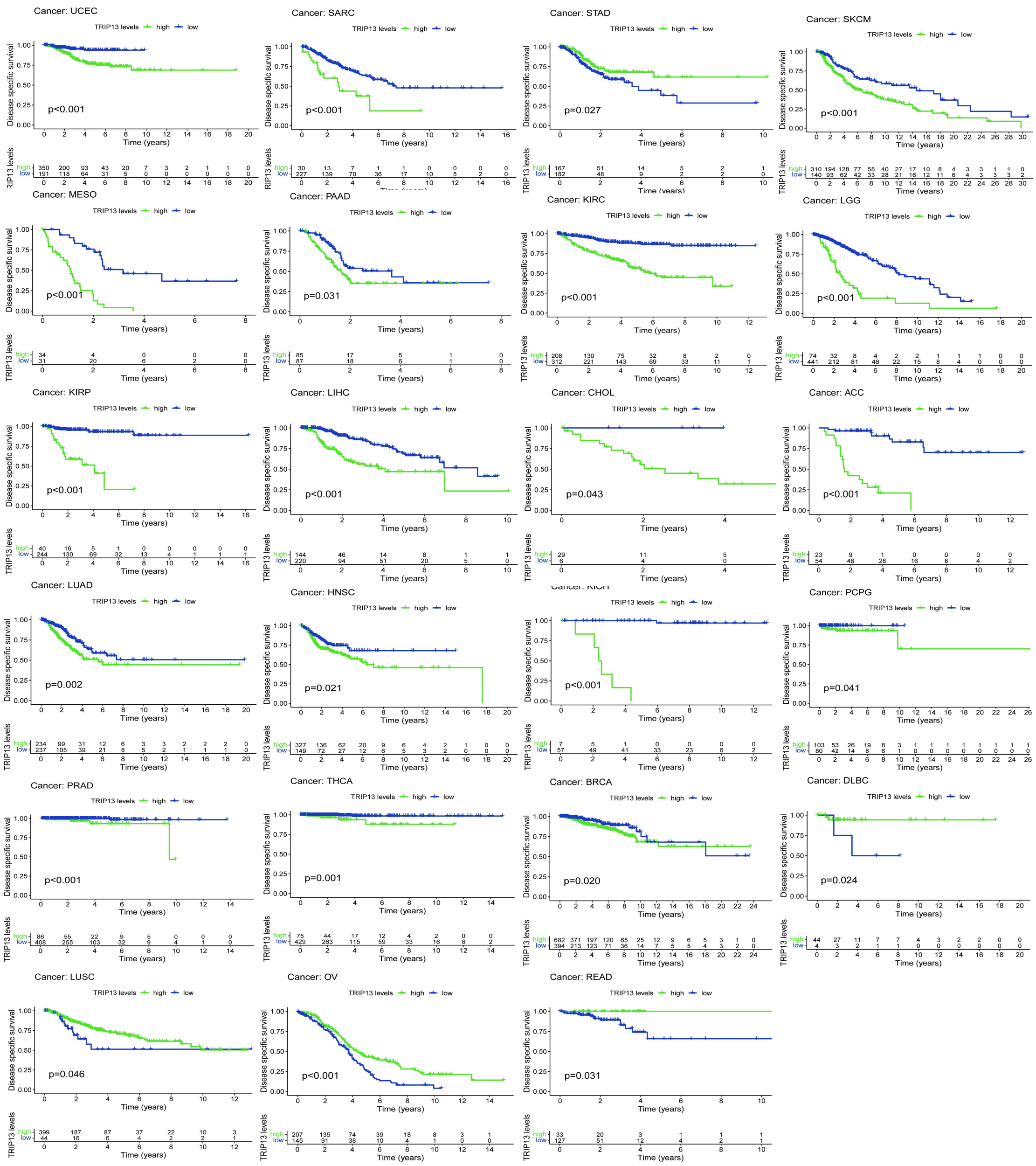

Supplementary figure 4. Predictive value of *TRIP13* expression in disease specific survival (DSS) in pan-cancer.

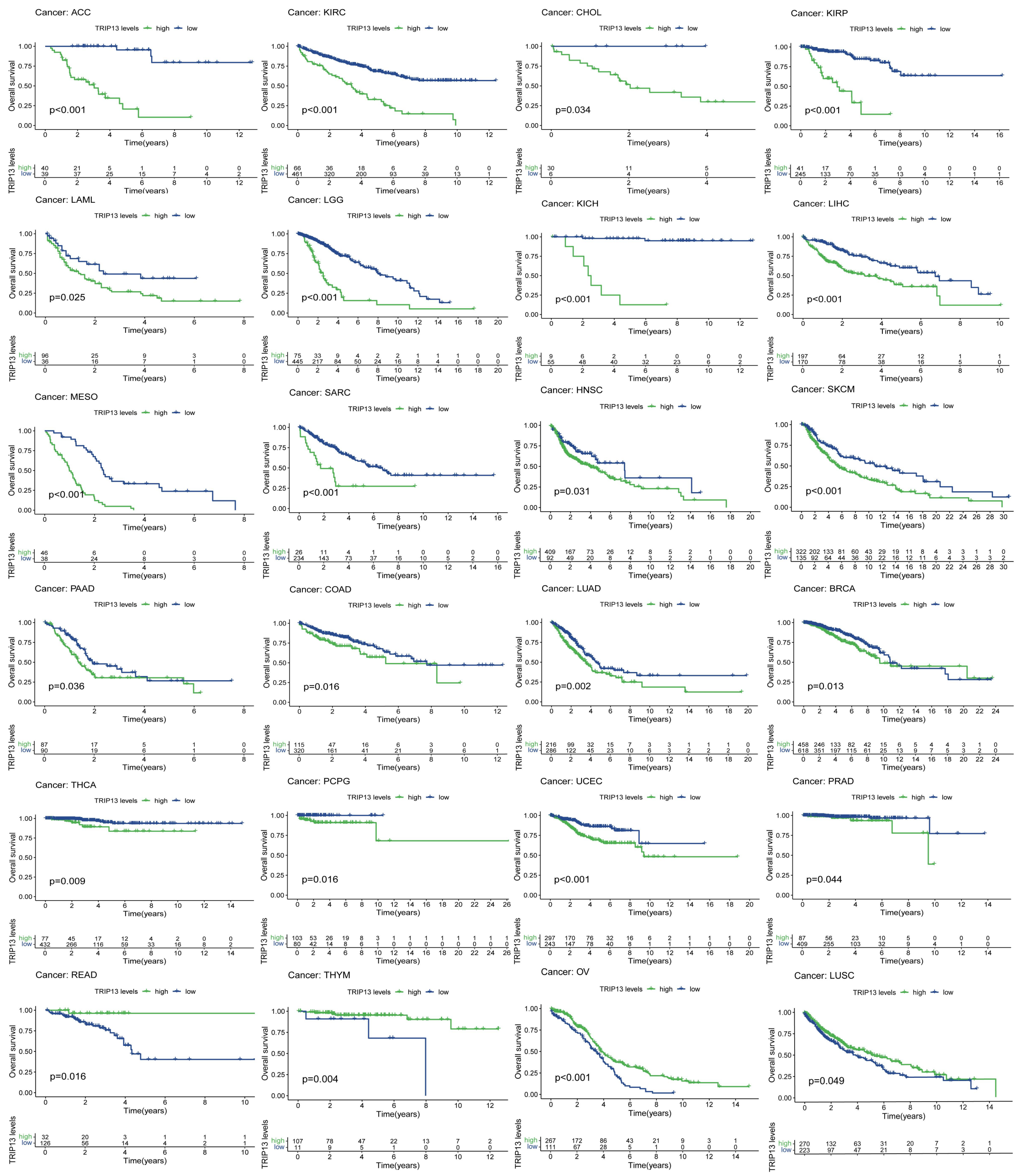

Supplementary figure 5. Predictive value of *TRIP13* expression in overall survival (OS) in pan-cancer.

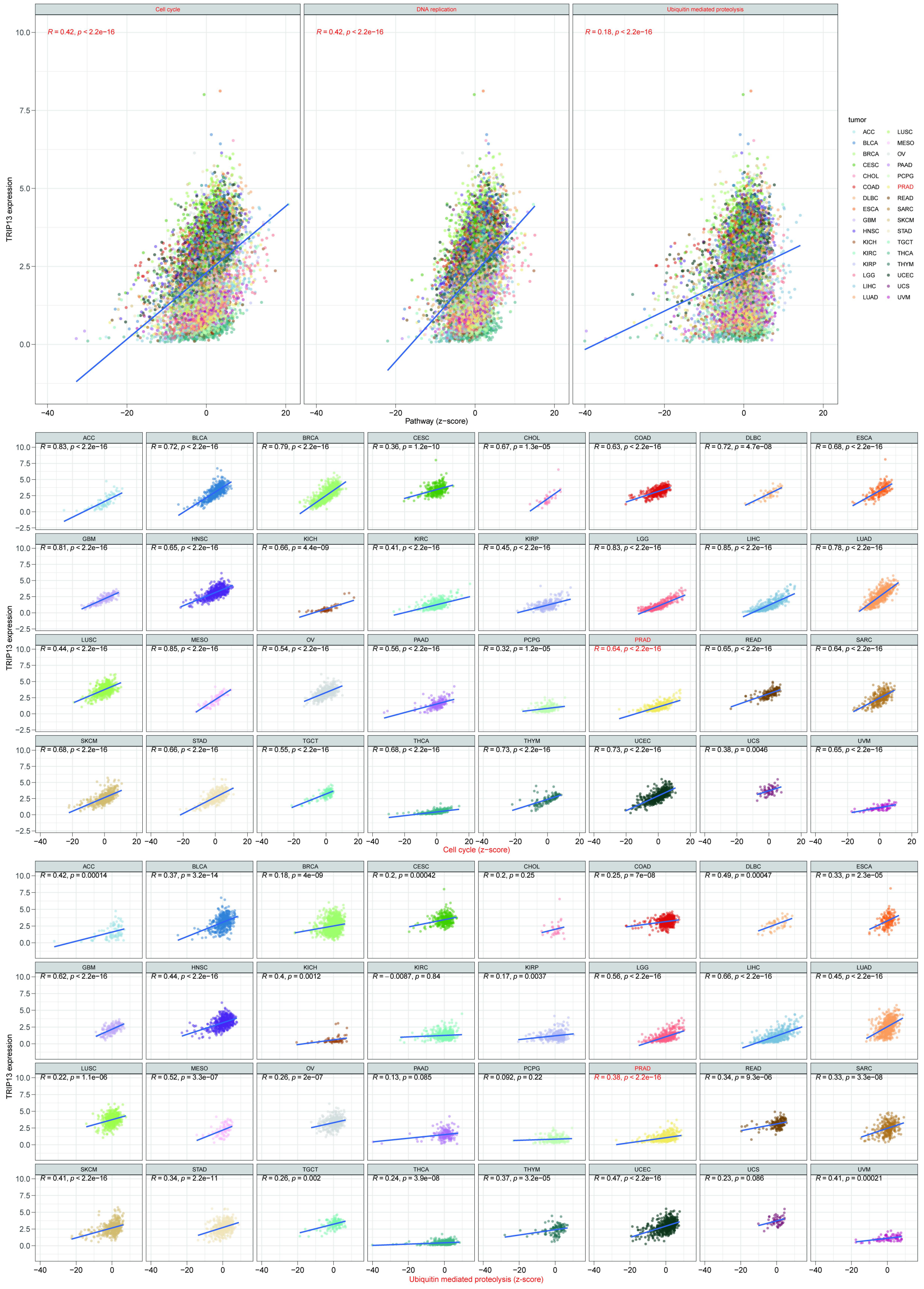

Supplementary figure 6. Correlation between *TRIP13* expression and pathway scores in multiple cancer types. Using the TCGA database, GSVA enrichment analysis was performed to calculate pathway enrichment scores across tumor samples. Correlation plots illustrate the relationship between *TRIP13* mRNA expression and enrichment scores for pathways including cell cycle, DNA replication, and ubiquitin-mediated protein proteolysis. Each data point represents an individual

sample, color-coded by tumor type, with regression lines depicting correlation trends. Spearman correlation coefficients (R) and corresponding p-values are indicated on each plot. The first row provides an overview of the correlation between *TRIP13* expression and the three pathways across all tumor types, while the subsequent rows detail the correlation of the cell cycle and ubiquitin-mediated protein hydrolysis pathways within individual cancer types.

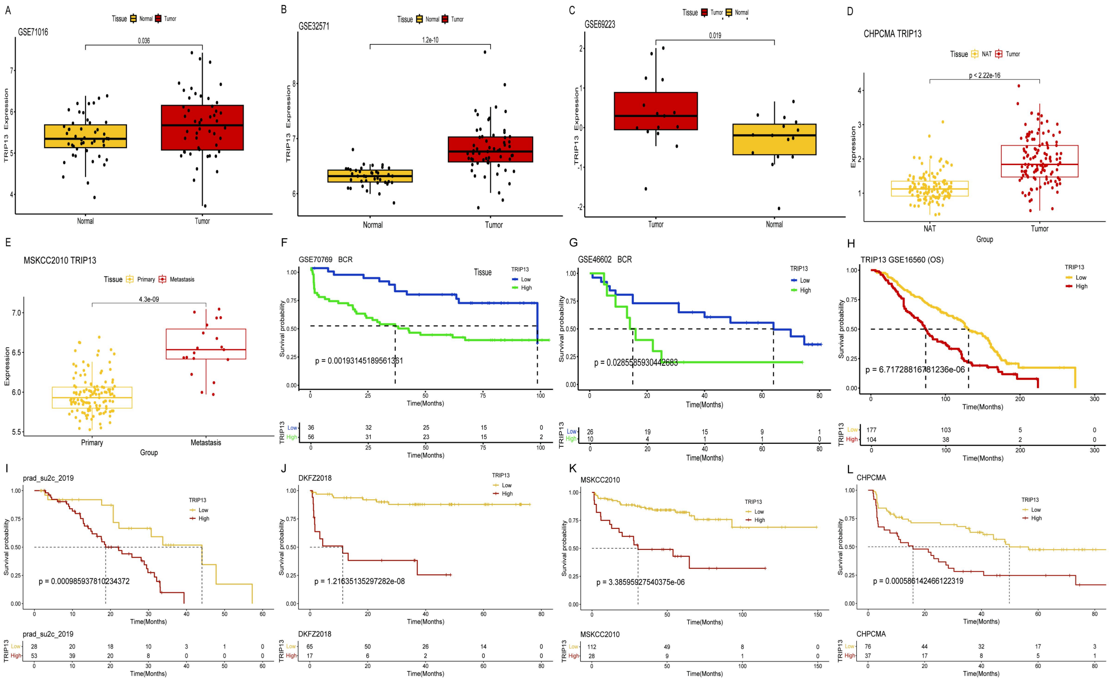

Supplementary figure 7. Validation of *TRIP13* expression in prostate cancer datasetss. (A-E) *TRIP13* exhibited significant elevation in tumor, with even higher levels observed in metastatic tissue. (F-L) Prostate cancer patients exhibiting high *TRIP13* expression experience poorer prognosis.

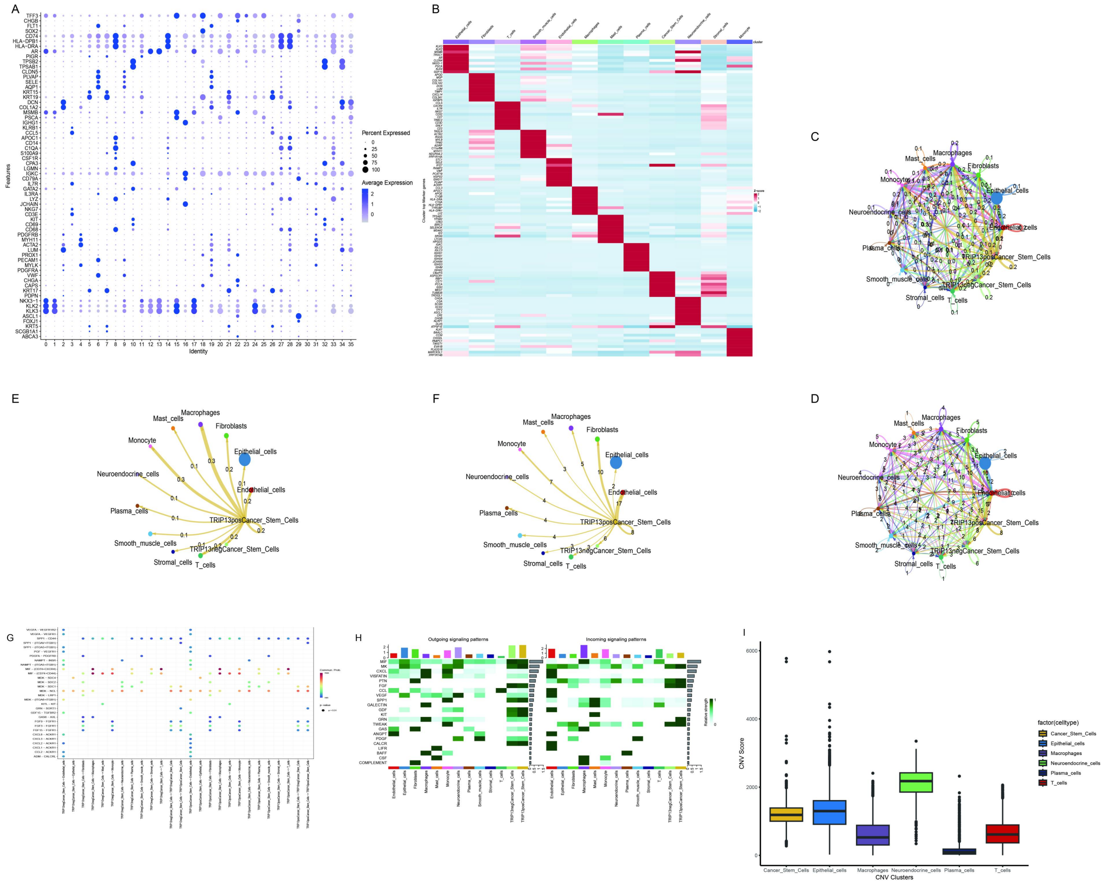

Supplementary figure 8. Single-cell transcriptome analysis. (A) Manually annotated single-cell dot plot. (B) Top 10 highly expressed genes in different cell types. (C-H) Cell to cell communication analysis using the CellChat R package. (I)Inference of copy number variation (CNV) using inferCNV revealed markedly elevated CNV levels in epithelial cells, tumor stem-like cells, and NEPC cells.

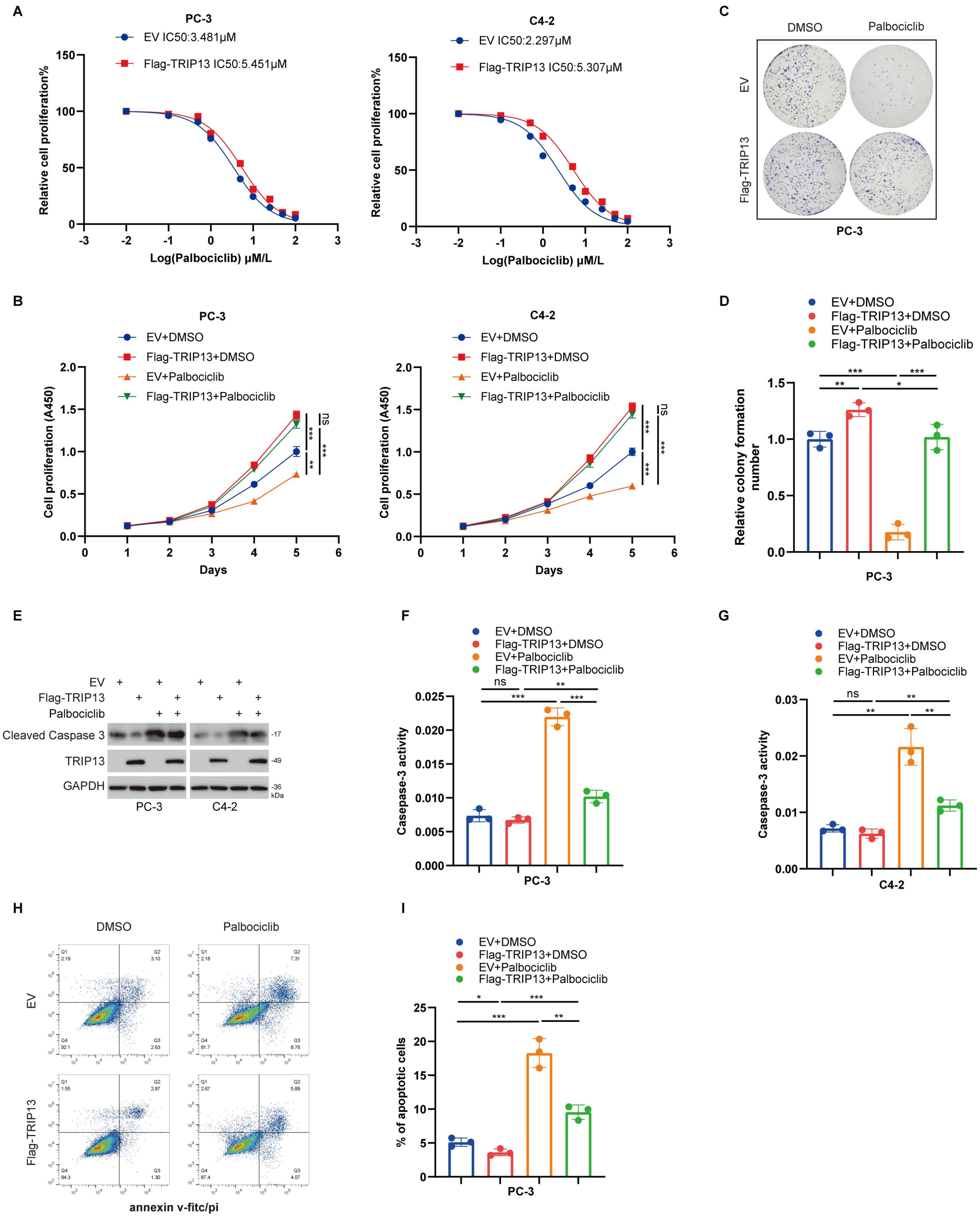

Supplementary figure 9. PC-3 and C4-2 cells were transfected with the specified plasmid for 24 hours, after puromycin screening, harvested cells were treated with continuous doses of palbociclib for 24 hours and harvested cells for CCK-8 assays to measure IC50 values of palbociclib (A). Data were expressed as mean ±SEM and repeated three times. (B) PC-3 and C4-2 cells were transfected with the specified plasmid for 24 hours. After puromycin selection, the cells were treated with or without Palbociclib (2 μM) for 24 hours and subsequently collected for the CCK-8 assay. Data were expressed as the mean ±SEM and repeated three times. ** indicates P < 0.01; *** indicates P < 0.001; ns, not significant. (C-D) PC-3 cells were transfected with a specified plasmid for 24 hours. After puromycin selection, the cells were treated with or without Palbociclib (2 μM) for 24 hours and subsequently

collected for colony formation assay. The data is displayed as mean ±SEM and repeated three times. * Indicates P < 0.05; ** indicates P < 0.01; *** indicates P < 0.001. (E-I) PC-3 and/or C4-2 cells were transfected with the specified plasmid for 24 hours. After puromycin selection, cells were treated with or without palbociclib (2 μM) for 24 hours. The cells were collected for Western blot analysis (E), caspase-3 activity assay (F-G) and Annexin V-FITC/PI assay (H-I). Data were expressed as mean ±SEM and repeated three times. * Indicates P < 0.05; ** indicates P < 0.01; *** indicates P < 0.001; ns, not significant.

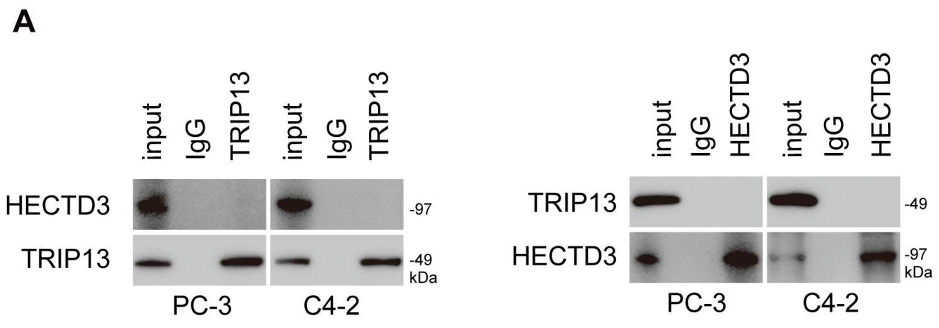

Supplementary figure 10. Immunoprecipitation analysis of the cell lysates of PC-3, or C4-2 cells by using the HECTD3 and TRIP13 antibodies (A).

Supplementary Table 1. The shRNA sequences.

shTRIP13 #1	5'-GATCCACTTCTAACATCACCGAGAA CTCGAGTTCTCGGTGATGTTAGAAGTGTTTTTG -3'
shTRIP13 #2	5'-GATCGCACTGTTGCACTTCACATTT CTCGAGAAATGTGAAGTGCAACAGTGCTTTTTG -3'
shE2F1 #1	5'-GATCCATCCAGCTCATTGCCAAGAA CTCGAGTTCTTGGCAATGAGCTGGATGTTTTTG -3'
shE2F1 #2	5'-GATCTAAGAGCAAACAAGGCCCGAT CTCGAGATCGGGCCTTGTTTGCTCTTATTTTTG -3'
shHECTD3 #1	5'-GATCTGCCGAGACTTTGCCAAGTAT CTCGAGATACTTGGCAAAGTCTCGGCATTTTTG -3'
shHECTD3 #2	5'-GATCGCAGTCTTCACCCAGGTATAT CTCGAGATATACCTGGGTGAAGACTGCTTTTTG -3'

Supplementary Table 2. The primer sequences for RT-qPCR.

Gene (Human)	Forward primer (5' - 3')	Reverse primer (5' - 3')
TRIP13	GAACACACAACCAGCAGACG	GGGAACCTTCTGTCATGCCT
HECTD3	CCGACATGGACCTACGAGTG	CACGTTGAACTCCTCCGTGT

Supplementary Table 3. The primer for ChIP-qPCR

Gene (Human)	Forward primer (5′ - 3′)	Reverse primer (5′ - 3′)
TRIP13	CTTTGTGCCTCTCTCGCTCA	CCACTGTGGTCTTCCCTTCC
